# Early modern human dispersal from Africa: genomic evidence for multiple waves of migration

**DOI:** 10.1186/s13323-015-0030-2

**Published:** 2015-11-06

**Authors:** Francesca Tassi, Silvia Ghirotto, Massimo Mezzavilla, Sibelle Torres Vilaça, Lisa De Santi, Guido Barbujani

**Affiliations:** Department of Life Sciences and Biotechnologies, University of Ferrara, Ferrara, Italy; Institute for Maternal and Child Health—IRCCS “BurloGarofolo”, University of Trieste, Trieste, Italy; Present Address: Leibniz Institute for Zoo and Wildlife Research, Berlin, Germany

**Keywords:** Human demographic history, Migration, Evolutionary divergence, Admixture, Linkage disequilibrium, Population structure

## Abstract

**Background:**

Anthropological and genetic data agree in indicating the African continent as the main place of origin for anatomically modern humans. However, it is unclear whether early modern humans left Africa through a single, major process, dispersing simultaneously over Asia and Europe, or in two main waves, first through the Arab Peninsula into southern Asia and Oceania, and later through a northern route crossing the Levant.

**Results:**

Here, we show that accurate genomic estimates of the divergence times between European and African populations are more recent than those between Australo-Melanesia and Africa and incompatible with the effects of a single dispersal. This difference cannot possibly be accounted for by the effects of either hybridization with archaic human forms in Australo-Melanesia or back migration from Europe into Africa. Furthermore, in several populations of Asia we found evidence for relatively recent genetic admixture events, which could have obscured the signatures of the earliest processes.

**Conclusions:**

We conclude that the hypothesis of a single major human dispersal from Africa appears hardly compatible with the observed historical and geographical patterns of genome diversity and that Australo-Melanesian populations seem still to retain a genomic signature of a more ancient divergence from Africa

**Electronic supplementary material:**

The online version of this article (doi:10.1186/s13323-015-0030-2) contains supplementary material, which is available to authorized users.

## Background

Anatomically modern humans (AMH), defined by a lightly built skeleton, large brain, reduced face, and prominent chin, first appear in the East African fossil record around 200,000 years ago [1, 2]. There is a general consensus that, while dispersing from there, they largely replaced preexisting archaic human forms [3]. Recent DNA studies also suggest that the replacement was not complete, and there was a limited, but nonzero, interbreeding with Neandertals [4], Denisovans [5], and perhaps other African forms still unidentified at the fossil level [6, 7]. As a result, modern populations might differ in the amount of archaic genes incorporated in their gene pool, which are eventually expressed and may result in phenotypic differences affecting, for example, the immune response [8] or lipid catabolism [9].

Although the general picture is getting clearer, many aspects of these processes are still poorly understood, starting from the timing and the modes of AMH dispersal. The main exit from Africa, through the Levant, has been dated around 56,000 years ago [10, 11]. However, morphologic [12, 13], archaeological [14], and genetic [13, 15–20] evidence suggest that part of the AMH population might have dispersed before that date, possibly by a Southern route into southern Asia through the horn of Africa and the Arab Peninsula.

Regardless of whether there was a single major expansion or two, several DNA studies clearly showed that genetic diversity tends to decrease [21, 22] and linkage disequilibrium to increase [23, 24] at increasing distances from Africa. This probably means that, as they came to occupy their current range, AMH went through a series of founder effects [25, 26]. These results offer an excellent set of predictions which we used in the present study to test whether current genomic diversity is better accounted for by processes involving a single major dispersal (hereafter: SD) or multiple major dispersals (hereafter: MD) from Africa.

One preliminary problem, however, is how to select the appropriate populations for informative comparisons. The details of the dispersal routes, and the relationships between fossils and contemporary populations, are all but established. Whereas Europeans are consistently regarded as largely derived from the most recent African exit in all relevant studies, opinions differ as for many aspects of the peopling of Asia [12–19], with many populations also experiencing complex demographic histories involving admixture, as suggested by both ancient [27] and modern [28–31] DNA evidence. To obtain insight into the past history of Eurasian populations, we analyzed genome-wide autosomal single nucleotide polymorphisms (SNPs) from 71 worldwide populations (Additional files 1 and 2). In what follows, a number of preliminary analyses allowed us to quantify the extent and the pattern of admixture and gene flow in our data, thus making it possible to identify a subset of Far Eastern populations which, under the MD model, may be regarded as largely deriving from the oldest expansion.

This way, we could tackle two questions, related, respectively, with the historical and geographical context of the dispersal process, namely (1) are separation times between non-African and African populations the same (as expected under SD), or is there evidence of a longer separation between Far Eastern and Africans than between Europeans and Africans (as expected under MD)? And (2) which geographical migration routes were followed by first humans outside Africa?

## Methods

### Populations and markers

Our analysis was based on public genomic datasets. No new biological sample was collected. We combined genomic data from several published datasets: the Human Genome Diversity Cell Line Panel [32] (*n* = 40 samples from 10 populations genotyped on Affymetrix GeneChip Human Mapping 500K Array Set), Pugach et al. (2013) [33] (*n* = 117 samples from 12 populations genotyped on an Affymetrix 6.0 array), Reich et al. (2009) [34] (*n* = 56 samples from 11 populations genotyped on an Affymetrix 6.0 array), Reich et al. (2011) [5] (*n* = 509 samples from 13 populations genotyped on an Affymetrix 6.0 array), Xing et al. (2009) [35] (*n* = 243 samples from 17 populations genotyped on one array (version NspI) from the Affymetrix GeneChip Human Mapping 500K Array Set), and Xing et al. (2010) [36] (*n* = 165 samples from 8 populations genotyped on an Affymetrix 6.0 array) (Additional files 1 and 2).

We devised a careful strategy to combine the seven datasets genotyped with different platforms according to different protocols developing a pipeline built on Perl. First, for each dataset, we checked for the presence of old rs IDs, if it is necessary changing them with the new ones. Then, we looked for the SNPs shared among all datasets, and we mapped the genome positions of these variants to the human reference genome, build hg18 (NCBI 36).

When merging data from different SNP chip versions, strand identification can be ambiguous, possibly leading to mistakes in identifying the right alleles for A/T and G/C SNPs (as also reported in the PLINK tool documentation [37]). Thus, to preserve as much genetic information as possible, we selected from each dataset only these ambiguous SNPs and we used the information contained in the Affymetrix Annotation file to evaluate the strand polarity used to define each allele. We considered each dataset separately and we annotated the SNPs on the plus strand, flipping only the proper SNPs. We checked the reliability of this conversion process comparing the allele frequencies for these SNPs in specific populations typed in more than one dataset (i.e., Besemah, CEU, Onge), so as to verify the consistency of the frequency spectrums between the different datasets. Once these ambiguities have been resolved, with the PLINK v 1.07 software [37] we merged progressively the datasets selecting, from each one, just the individuals from populations of our interest and flipping SNPs discordant for strand.

**Fig. 1:**
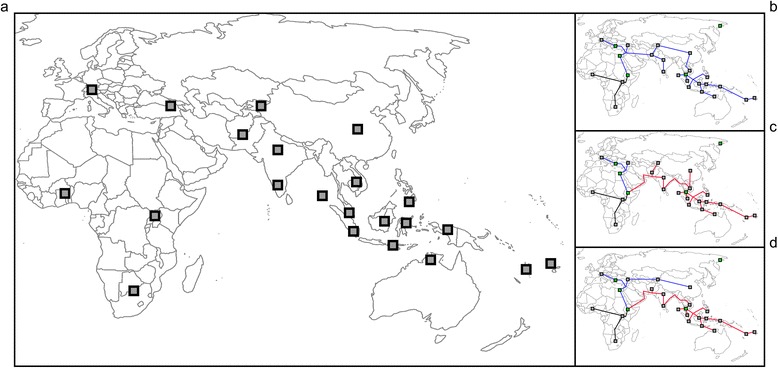
Geographic location of the 24 metapopulations analyzed (**a**) and geographical models of African dispersal (**b**, **c**, **d**). Metapopulations, each derived from the merging of genomic data from several geographically or linguistically related populations, are South, East, and West Africa; Europe; Caucasus; South, East, West, and Central Asia; and North and South India, plus three Negrito (Onge, Jehai, and Mamanwa) and ten Oceanian populations; the final dataset comprised 1130 individuals. Under model 1, a SD model (**b**), all non-African populations are descended from ancestors who left Africa through the same northern route [3]. Model 2 (**c**) and model 3 (**d**) are MD models assuming, prior to dispersal across Palestine, another exit through the Arab Peninsula and the Indian subcontinent; under model 2, all Asian and Western Oceanian populations derive from this earlier expansion [12], whereas under model 3 only the populations of Southeast Asia and Western Oceania derive from the earlier expansion [16]

Using the same software, we selected only the autosomal SNPs with genotyping success rate >98 % and minor allele frequency (MAF) >0.01. We identified cryptic relatedness among samples computing identity by descent (IBD) statistic for all pairs of individuals, as unmodeled excess of genetic sharing would violate sample independence assumption of downstream analyses. When pairs of individuals showed a Pi-Hat value >0.3, we removed the individual with the lowest genotyping rate. We did not apply this screening procedure for the Southeast Asia and Oceania samples, since they come from populations with extremely low effective sizes, where a certain degree of random inbreeding is inevitable [38]. To determine whether there were genetic outliers within each population, we conducted in PLINK a “distance to the nearest neighbor analysis” (neighbor option). Within each population, the measure of similarity in terms of identity by state (IBS) between each individual and their nearest neighbor was calculated and transformed into a *Z*-score. *Z*-score distributions were examined from the first to the fifth neighbor. Outliers were identified by an extremely negative *Z*-score produced by *low* allele sharing with their nearest neighbor and were then dropped from the population. We grouped populations according to ethnological and linguistic information; the final dataset is shown in Fig. 1a.

To visualize the genetic relationships between such populations, we performed a principal component analysis using the R [39] SNPRelate package.

### Population structure analysis

Individual genotypes were clustered, and admixture proportions were inferred, by the algorithm embedded in the software ADMIXTURE, based on the principle of maximum likelihood [40]. This method considers each genotype as drawn from an admixed population with contributions from *k* hypothetical ancestral populations. Because this model assumes linkage equilibrium among markers, we checked with the PLINK v1.07 tool [37] that the set of SNPs we used did not show a level of linkage disequilibrium higher than *r*^2^ = 0.3; this way, in the pruned dataset, 54,978 markers were retained. The optimal value of *k* was evaluated through a cross-validation procedure, testing values from *k* = 2 to *k* = 14, thus identifying the number of ancestral populations for which the model had the best predictive accuracy. We then ran an unsupervised analysis, assuming a number of ancestral admixing populations from *k* = 2 to *k* = 7. The proportion of the individuals’ genome belonging to each ancestral population was calculated for each *k* value from five independent runs, then combined by the software CLUMPP [41] and plotted by the software *Distruct* [42].

### Discriminant Analysis of Principal Components

In addition to ADMIXTURE, to identify and describe clusters of genetically related individuals, we used a Discriminant Analysis of Principal Components (DAPC) [43] implemented in the R [39] package adegenet ver. 1.3-9.2 [44]. DAPC methods allow one to assess the relationships between populations overlooking the within-group variation and summarizing the degree of between-group variation. Being a multivariate method, DAPC is suitable for analyzing large numbers of genome-wide SNPs, providing assignment of individuals to different groups and an intuitive visual description of between-population differentiation. Because it does not rely on any particular population genetics model, DAPC is free of assumptions about Hardy-Weinberg equilibrium or linkage equilibrium [43], and so we could use the full set of 96,156 SNPs for this analysis.

By the function *find.clusters*, we determined the most likely number of genetic clusters in our dataset, using all principal components (PCs) calculated on the data. The method uses a K-means clustering of PCs [10] and a Bayesian information criterion (BIC) approach to assess the best supported number of clusters.

Then, we determined the optimal number of PCs to retain to perform a discriminant analysis avoiding unstable (and improper) assignment of individuals to clusters. It is worth noting that, unlike K-means, DAPC can benefit from not using too many PCs: retaining too many components with respect to the number of individuals can lead to over-fitting and instability in the membership probabilities returned by the method.

### Population divergence dates

The divergence times between populations (*T*) were estimated from the population differentiation index (*F*_ST_) and the effective population size (*N*_e_). *F*_ST_ is the proportion of the total variance in allele frequencies that is found between groups, and it was calculated between pairs of populations for each SNP individually under the random population model for diploid loci, as described by Weir and Cockerham [45], and then averaged over all loci to obtain a single value representing pairwise variation between populations. Under neutrality, the differences between populations accumulate because of genetic drift, and so their extent depends on two quantities: it is inversely proportional to the effective population sizes (*N*_e_) and directly proportional to the time passed since separation of the two populations (*T*).

Therefore, to estimate *T* from genetic difference between populations, independent estimate of *N*_e_ is needed; for this purpose, we focused on the relationship between *N*_e_ and the level of linkage disequilibrium within populations. Indeed, levels of linkage disequilibrium (LD) also depend on *N*_e_ and on the recombination rate between the SNPs considered [46], with LD between SNPs separated by large distances along the chromosome reflecting the effects of relatively recent *N*_e_, whereas LD over short recombination distances depending on relatively ancient *N*_e_ [47]. Thus, we estimated LD independently in each population using all polymorphic markers available for that population (MAF > 0.05), from a minimum of ~90,000 SNPs in Polynesia to a maximum of ~370,000 SNPs in North India. This way, we also reduced the impact of ascertainment bias, i.e., the bias due to the fact that most SNPs in the genotyping platforms were discovered in a single (typically European) population [48]. This approach was already followed by McEvoy et al. in 2011 [18] to estimate the divergence times of diverse non-African populations from Africa; however, in this study we extended that the genomic data in the analysis to incorporate populations that previous work has suggested are ancestrally related to an earlier exit from Africa, i.e., “relic” populations from Southeast Asia [20].

We assigned to each SNP a genetic map position based on HapMap2 (Release #22) recombination data, and for each pair of SNPs separated by less than 0.25 cM, we quantified LD as *r*^2^_LD_ [49] or as *σ*^2^_LD_ [50] (hereafter: *ρ*). All the observed *ρ* values were then binned into one of the 250 overlapping recombination distance classes. Pairs of SNPs separated by less than 0.005 cM were not considered in the analysis, since at these very short distances, gene conversion may mimic the effects of recombination [46]. We also adjusted the *ρ* value for the sample size using $\rho -\left({\scriptscriptstyle \frac{1}{n}}\right)$ [46]. Finally, we calculated the effective population size for each population in each recombination distance class as

$$ {N}_{\mathrm{e}}\kern0.5em =\kern0.5em \left({\scriptscriptstyle \frac{1}{4c}}\right)\kern0.5em \left[{\scriptscriptstyle \frac{1}{\rho }}-2\right], $$

corresponding to the effective population size $\frac{1}{2}C$ generations ago, where *c* is the recombination distance between loci, in Morgans [47, 51, 52]. Finally, the long-term *N*_e_ for each population was calculated as the harmonic mean of *N*_e_ over all recombination distance classes up to 0.25 cM. The confidence intervals of these *N*_e_ values were inferred from the observed variation in the estimates across chromosomes.

Based on the independently estimated values of *N*_e_, we could then estimate *T* as $T\kern0.5em =\kern0.5em  \ln \kern0.5em \left(1-{F}_{ST}\right)/ ln\left(1-\left({\scriptscriptstyle \frac{1}{2{N}_e}}\right)\right)$ [53] where time is expressed in generations.

All procedures were performed by in-house-developed software packages, NeON [54] and 4P [55].

### Simulations

To understand whether the divergence times estimated were compatible with a SD model, we used a neutral coalescent approach to simulate genetic polymorphism data under the infinite sites model of mutation. We simulated data representing 1-Mb chromosome segments in two populations according to the demographic scenario shown in Additional file 3a using the coalescent simulator *ms* [56]. We assumed an ancestral population with an initial *N*_e_ = 10,000. At *t* = *T*, the population splits into two populations. Population_2a’s *N*_e_ remains constant; population_2b has a 50 % reduction in *N*_e_ followed by an exponential growth, representing the genetic bottleneck experienced by populations dispersing out of Africa. In all simulations, the scaled mutation rate (*θ*) and the scaled population recombination rate (*ρ*) were fixed at 400. For a sequence length of 1 Mb and an effective population size of *N*_e_ = 10,000, these parameters correspond to a mutation rate of 10^−8^ and a recombination rate of 1 cM/Mb.

To account for the uncertainty in the estimates of both the timing of the process and the effective population sizes, according to [57–59], this model was simulated considering 4 different separation times (*T*) (between 40,000 and 70,000 years ago, in steps of 10,000 years) and 6 estimates of the actual effective size for population_2b (between 3000 and 8000, in steps of 1000). For each of the 24 simulation conditions, 1000 independent datasets were simulated and then analyzed according to the following procedure:

1. A sample of 50 individuals (i.e., 100 chromosomes) was randomly selected from each population. The simulated genetic data were single nucleotide polymorphisms (SNPs) segregating within the two populations.
2. We converted the ms [56] output file to PLINK format [37].
3. Any SNPs with a MAF less than 0.05 were removed from the datasets.
4. We estimated the population differentiation index, effective population size, and divergence times between the two simulated populations following the same procedures used for the observed data and detailed above.
5. Estimators were calculated for each 1000 independent replications.

For a subset of combinations of parameters (effective population size of population_2b = 3000 and the whole range of possible divergence times), we considered a more complicated (and more realistic) model that includes several founder effects after the dispersal from Africa. We simulated a founder effect every 500 generations (for a total of three events), each time generating a reduction of the 50 % of the populations, followed by an exponential growth (Additional file 3 b). For each combination of parameters, we simulated 5000 independent datasets, then analyzed as detailed above.

### Possible effects of a Denisovan admixture in Melanesia

To rule out the possibility that the divergence times estimated between Africans and New Guinea/Australia samples could reflect, largely or in part, admixture between the Denisovan archaic human population from Siberia [60] and the direct ancestor of Melanesians, we removed from our dataset the variants that could be regarded as resulting from such a process of introgression. These SNPs would carry the derived state in the archaic population and in the New Guinean/Australian samples, while being ancestral in East Africans and Europeans (i.e., those populations that did not show any signal of introgression from Denisova [5, 60]).

Using the *VCFtools* [61], we extracted our 96,156 SNPs from the high-coverage Denisovan genome. We then removed from these data all transition SNPs (C/T and G/A) because in ancient DNA, these sites are known to be prone to a much higher error rate than the transversions [5]. Then, we selected the sites meeting the following set of criteria:

- The site has human-chimpanzee ancestry information.
- The human-chimpanzee ancestral allele matches one of the two alleles at heterozygous sites.
- Denisova has at least one derived allele, New Guineans and Australians have at least one derived allele, and the ancestral allele is fixed in East African and European individuals.
- Denisova has at least one ancestral allele, New Guineans and Australians have at least one ancestral allele, and the derived allele is fixed in East African and European individuals.

When the ancestry information was missing (1438 SNPs), to define the ancestral state, we used the East African individuals selecting the SNPs where East Africans were homozygous and considering those as ancestral.

Once we had thus identified a subset of sites putatively introgressed from Denisova, we removed them from the dataset. The remaining 80,619 SNPs were used to compute the pairwise *F*_ST_ [45] values and to infer the divergence times between populations, as described above.

### Testing for the effect of recent gene flow

Using *TreeMix*, we inferred from genomic data a tree in which populations may exchange migrants after they have split from their common ancestor, thus violating the assumptions upon which simple bifurcating trees are built [62]. This method first infers a maximum likelihood tree from genome-wide allele frequencies and then identifies populations showing a poor fit to this tree model; migration events involving these populations are finally added. This way, each population may have multiple origins, and the contributions of each parental population provide an estimate of the fraction of alleles in the descendant population that originated in each parental population.

Allele frequencies for the TreeMix analysis were calculated by PLINK tool [37], after pruning for LD as we did for ADMIXTURE analysis. We modeled several scenarios allowing a number of migration events from zero to six and stopping adding a migration when the following event did not increase significantly the variance explained by the model. The trees were forced to have a root in East Asia, and we used the window size of 500 (−*k* option).

We also ran a three-population test, calculating an *f*_3_ statistic [34] for all population triplets by software threepop of the TreeMix package [62].

### Geographical patterns of dispersal

We developed explicit geographic models of demographic expansion and looked for the model giving the closest association between genomic and geographical distances. In all cases, migration routes were constrained by five obligatory waypoints, identified in [26] and accepted by several successive studies (see, e.g., [13]). In addition, because of some inconsistencies in the definition of the geographic regions affected by the two waves of migration under MD [12, 14, 16], and of the ambiguity introduced by the previously described episodes of admixture, two different models of MD were considered. Under model 1, a SD model, AMH left Africa through Palestine and dispersed into both Europe and Asia (Fig. 1b). Model 2 assumes, prior to the dispersal across Palestine, another exit through the Arab Peninsula and the Indian subcontinent, all the way to Melanesia and Australia; according to this model, based on skull morphology [12] all Asian populations are derived from this earlier expansion (Fig. 1c). On the contrary, under model 3 only the populations of Southeast Asia and Oceania are derived from the earlier expansion, whereas Central Asian populations are attributed to the later African dispersal [16] (Fig. 1d).

To obtain a realistic representation of migrational distances between populations, we did not simply estimate the shortest (great circle) distances between sampling localities. Rather, we modeled resistance to gene flow, based on the landscape features known to influence human dispersal. We used a resistance method from the circuit theory implemented in the software Circuitscape v.3.5.2 [63], starting from the landscape information in [64] and referring to the distribution of land masses at the last glacial maximum. Next, we added data about altitude and river presence from the Natural Earth database. Each area of the map was assigned a resistance value (rv) by the Reclassify tool in ArcGIS 10 (ESRI; Redlands, CA, USA), as follows: mountains higher than 2000 m: rv = 100; land or mountains below 2000 m: rv = 10; rivers: rv = 5; oceans: NoData (absolute dispersal barrier); and narrow arms of sea across which prehistoric migration is documented: rv = 10. The low rv for rivers reflects the human tendency to follow, whenever possible, water bodies in their dispersal (see, e.g., [65]).

Under the SD model, we hampered movement from Arabia to India (rv = 100), hence preventing the dispersal along the Southern route; under the MD models, we created a buffer of low resistance value (rv = 1) along the Southern route. For all models, we then estimated least-resistance distances between the populations analyzed, when applicable going through Addis Ababa, chosen as a starting point for the African expansion [26]. The final output was then exported in Google Earth where geographic distances were expressed in kilometers.

We evaluated by partial Mantel tests [66] the correlation between genomic (*F*_ST_) and geographic distances, while holding divergence times constant. This way, we could control for the drift effects, due to the fact that populations separated at distinct points in time and space.

## Results

### Genomic structure of Old World populations

We assembled genome-wide SNP data from the literature obtaining information on 71 population samples sharing, after cleaning and integration, 96,156 autosomal SNPs. By merging samples from adjacent geographical regions and with similar linguistic affiliations, we organized the data in 24 metapopulations; the final dataset comprised 1130 individuals (Fig. 1a and Additional file 2).

**Fig. 2:**
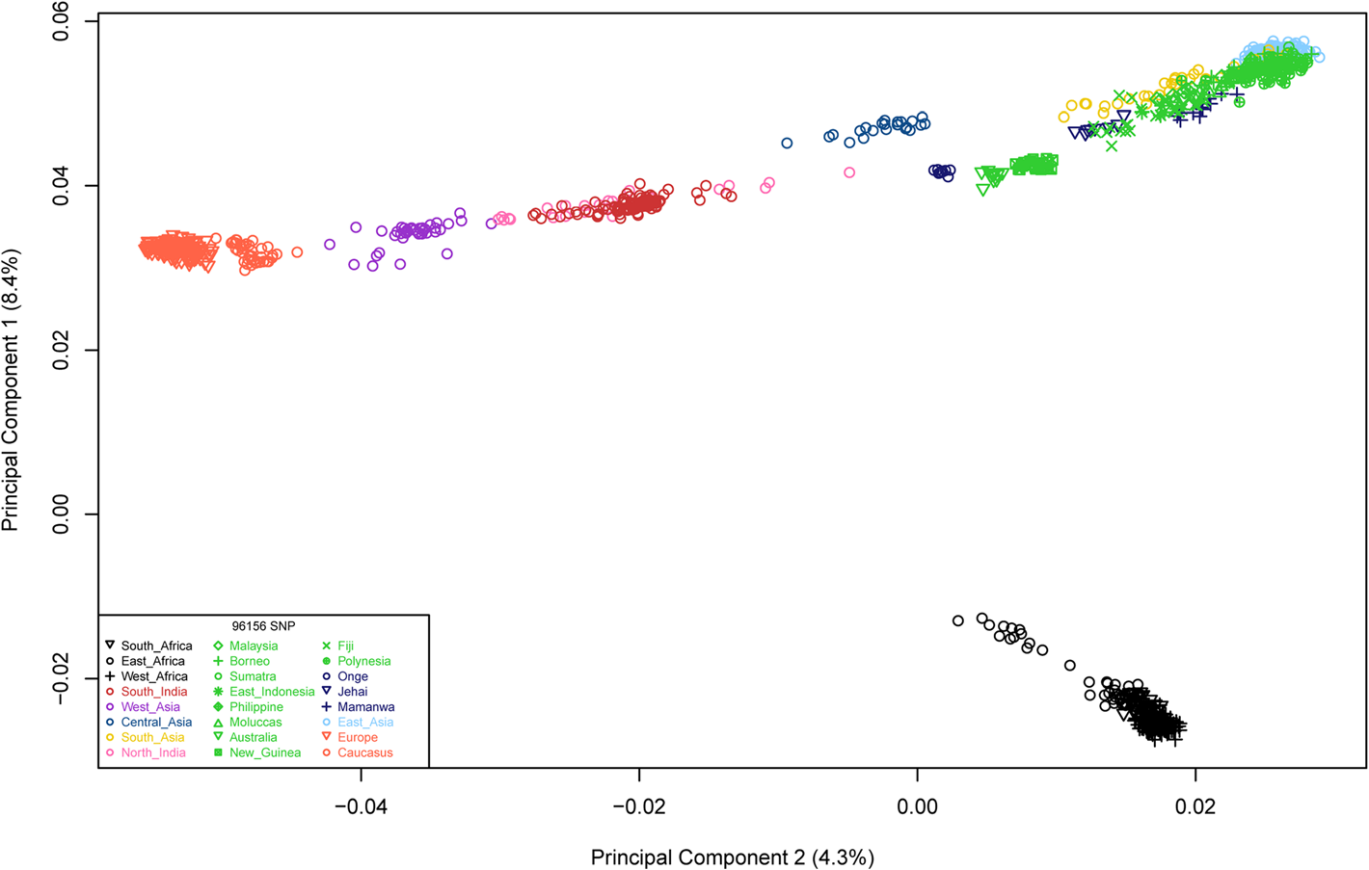
Results of the principal component analysis. Each symbol corresponds to an individual genotype; the first two principal components account for 12.7 % of the global variation in the data. Here and in all figures, *different colors* represent different geographical regions

As a preliminary step, we visualized by principal component analysis the genetic relationships between such populations, as inferred from these autosomal SNPs (Fig. 2). The first two PCs, accounting respectively for 8.4 and 4.3 % of the total genetic variance, show that the populations we grouped in metapopulations do cluster together genetically. In addition, genetic relationships largely correspond to geographical distances, with Eurasian populations separated from the African ones along the axis represented by PC1, and forming an orderly longitudinal cline, all the way from Europe to East Asia and Oceania, along the PC2 axis.

**Fig. 3:**
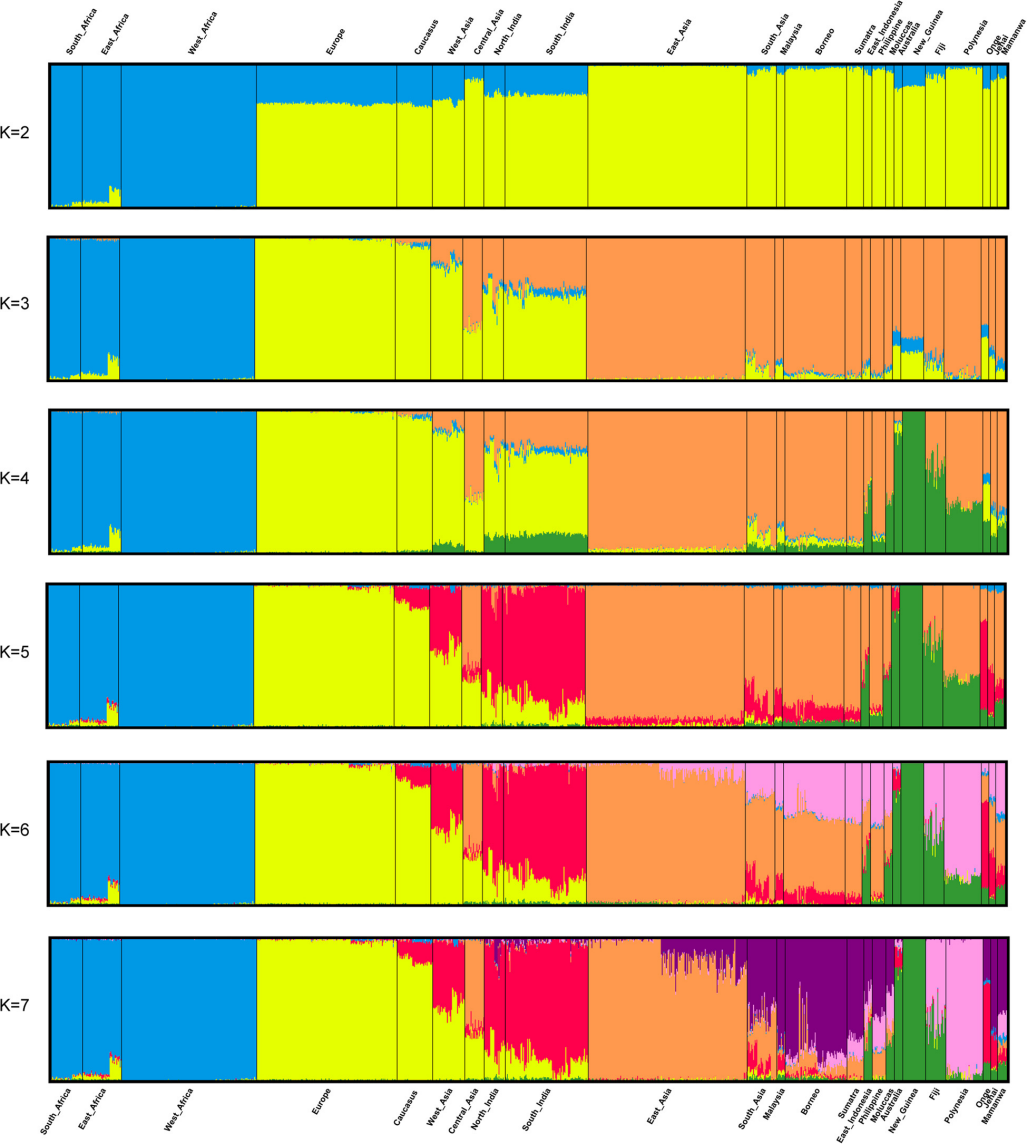
ADMIXTURE analysis of 1130 individuals in 24 populations from Africa, Eurasia, and Western Oceania. Each individual genotype is represented by a *vertical column*, the *colors* of which correspond to the inferred genetic contributions from *k* putative ancestral populations. The analysis was run for 2 ≤ *k* ≤ 7

Then, to further investigate the worldwide genomic structure, we applied the unsupervised ancestry inference algorithm of the ADMIXTURE software [40]. After identifying *k* = 6 as the most supported number of ancestral populations (Additional file 4), we explored the results for *k* = 2–7 ancestral populations. As the number of ancestral clusters increased, we observed the emergence of several well-supported population-specific ancestry clusters (Fig. 3). At *k* = 2, the ancestry assignment differentiated between African (blue) and non-African (yellow) populations; *k* = 3 further distinguishes Europeans from Asians (orange); *k* = 4 identifies an Australo-Melanesian component (green) within the Asian cluster; at *k* = 5, the additional component is mainly associated with the Indian subcontinent (red); the same is the case at *k* = 6 for Polynesia and Fiji (pink) and at *k* = 7 for many island communities of Southeast Asia and Oceania (purple). Remarkably, some populations show more than 99 % contribution from the same ancestral population along different *k* values (e.g., West Africa, Europe, New Guinea), whereas other populations include several individuals with an apparently admixed genomic background, possibly resulting from successive gene flow (e.g., back migration from Europe to Northeast Africa [67]). A DAPC [43] led to essentially the same conclusions as ADMIXTURE. We found *k* = 6 to be the best supported model (Additional file 5) and therefore used this value in the DAPC. Additional file 6a shows that the main populations are distinguishable, and most individuals from the same population tend to fall in the same cluster. In the scatterplot, the first two axes revealed three major clusters within the six supported by the *k* = 6 model (Additional file 6b). They included (i) the three African populations, (ii) most populations from Asia, and (iii) populations from Europe and Caucasus and from India and West Asia. This clustering pattern is also observed in ADMIXTURE analysis with *k* = 6 (Fig. 3). Interestingly, in the Asian group the DAPC is able to distinguish three different clusters: one represented by individuals from Australia and New Guinea (in green color), one by the populations showing at least 30 % of the green ADMIXTURE component at *k* = 5 (in pink color), and one by other populations from Asia.

### Population divergence dates

There is a clear geographical structure in the data, which in principle allows one to test for the relative goodness of fit of the two models. The SD model implies that the separation time from Africa of all samples should be the same, whereas significantly larger times of separation are expected under the MD model for the eastern most than for the European populations. To answer, we calculated the divergence times between each out-of-Africa population and East, South, and West African populations as described in the “Methods” section, obtaining an independent estimate of *N*_e_ from LD which allowed us to tell apart the effects of population sizes and separation times in the observed *F*_ST_ values.

The three African populations show the largest long-term population sizes (calculated as the harmonic mean of *N*_e_ values) and a constant declining trend through time, whereas Eurasian populations (and more markedly the Asian ones) tend to increase in size, especially in the last 10,000 years. Australians and New Guineans (represented in green in the ADMIXTURE analysis at *k* ≥ 4) generally maintain a constant size until present times, whereas the Negrito populations show low and declining sizes. In general, these results were not surprising, but the fact we obtained them suggests that the procedure followed is by and large accurate and therefore that the estimated average *N*_e_s are plausible. The values obtained using the two estimators of LD (*r*^2^ and *σ*^2^, see “Methods” section) gave similar results (Additional files 7 and 8).

From the pairwise *F*_ST_ values estimated over all loci (Additional file 9), and now considering the independently estimated values of *N*_e_, we could infer the divergence times between populations (Table 1 and Additional file 10). The average separation times from the East African populations, i.e., those located in the most plausible site of departure of AMH expansions [26] (Table 1), are distributed along a range spanning from 60K to 100K years ago. Extreme divergence values were observed for Europe and Caucasus on the one hand, and for Australia and New Guinea on the other, respectively, at the lower and the upper tails of the distribution. Even considering the full range of uncertainty around these estimates (95 % of the confidence interval), we observed no overlap, with Europe having an upper confidence limit 77K/71K years ago (depending on the LD measure used, respectively, the *r*^2^ and *σ*^2^ statistic) and Australia having a lower confidence limit 88K/80K years ago. The harmonic means of the effective population sizes we estimated were not correlated with the divergence times, meaning that this difference cannot possibly be accounted for by the different impact of genetic drift upon these populations. Therefore, this result supports a rather complex “out-of-Africa” scenario, suggesting at least two main phenomena of AMH dispersal from Africa. The Australo-Melanesian populations, i.e., Australians and New Guineans, with their early separation times from East Africa, may be regarded as the putative descendants of an early dispersal process, whereas the status of most Asian populations would seem, at this stage of the analysis, unclear.

**Table 1:** Estimated divergence times from (East) Africa using the *r* ^2^ or *σ* ^2^ statistics as estimators of LD level

Time	*r* ^2^ [49]			*σ* ^2^ [50]		
Population	Lower 95% confidence limit	Point estimate	Upper 95% confidence limit	Lower 95% confidence limit	Point estimate	Upper 95% confidence limit
	0.025	0.5	0.975	0.025	0.5	0.975
Europe	62,664	69,736	78,916	56,406	65,402	74,773
Caucasian	60,080	68,143	76,336	54,193	63,196	70,825
West_Asia	60,187	66,318	74,087	53,903	61,586	68,741
Central_Asia	65,785	71,021	80,083	59,337	66,425	75,228
North_India	65,533	70,230	79,438	59,671	65,243	74,008
South_India	59,931	64,396	71,782	54,307	60,664	68,214
East_Asia	80,536	87,432	97,802	72,917	81,398	91,230
South_Asia	73,452	80,587	89,791	66,565	74,425	83,899
Malaysia	66,345	71,622	81,344	60,177	66,852	75,702
Borneo	74,750	80,056	89,253	67,372	74,579	84,318
Sumatra	75,306	82,043	91,758	68,310	76,108	85,344
East_Indonesia	66,578	71,576	81,948	60,417	67,056	75,495
Philippine	73,171	79,248	89,786	66,592	73,996	84,524
Moluccas	66,115	71,562	80,897	60,106	66,875	75,578
Australia	87,017	96,599	111,394	78,167	89,596	102,557
New_Guinea	98,962	107,204	121,010	88,631	99,499	113,990
Fiji	71,173	77,395	85,605	64,780	72,155	80,833
Polynesia	71,230	77,531	87,551	64,808	72,451	81,800
Onge	76,885	82,572	92,571	69,842	77,670	87,794
Jehai	65,885	71,521	80,870	59,903	66,859	76,395
Mamanwa	67,671	73,012	83,689	61,558	68,502	78,951

For each comparison with East Africa, the three columns report the 95 % lower confidence limit, the point estimate (in years, assuming a generation interval = 25 years, as proposed by [75]), and the 95 % upper confidence limit

For each comparison with East Africa, the three columns report the 95 % lower confidence limit, the point estimate (in years, assuming a generation interval = 25 years [88]), and the 95 % upper confidence limit

### Comparing the predictions of single vs multiple African exit models: divergence times

**Fig. 4:**
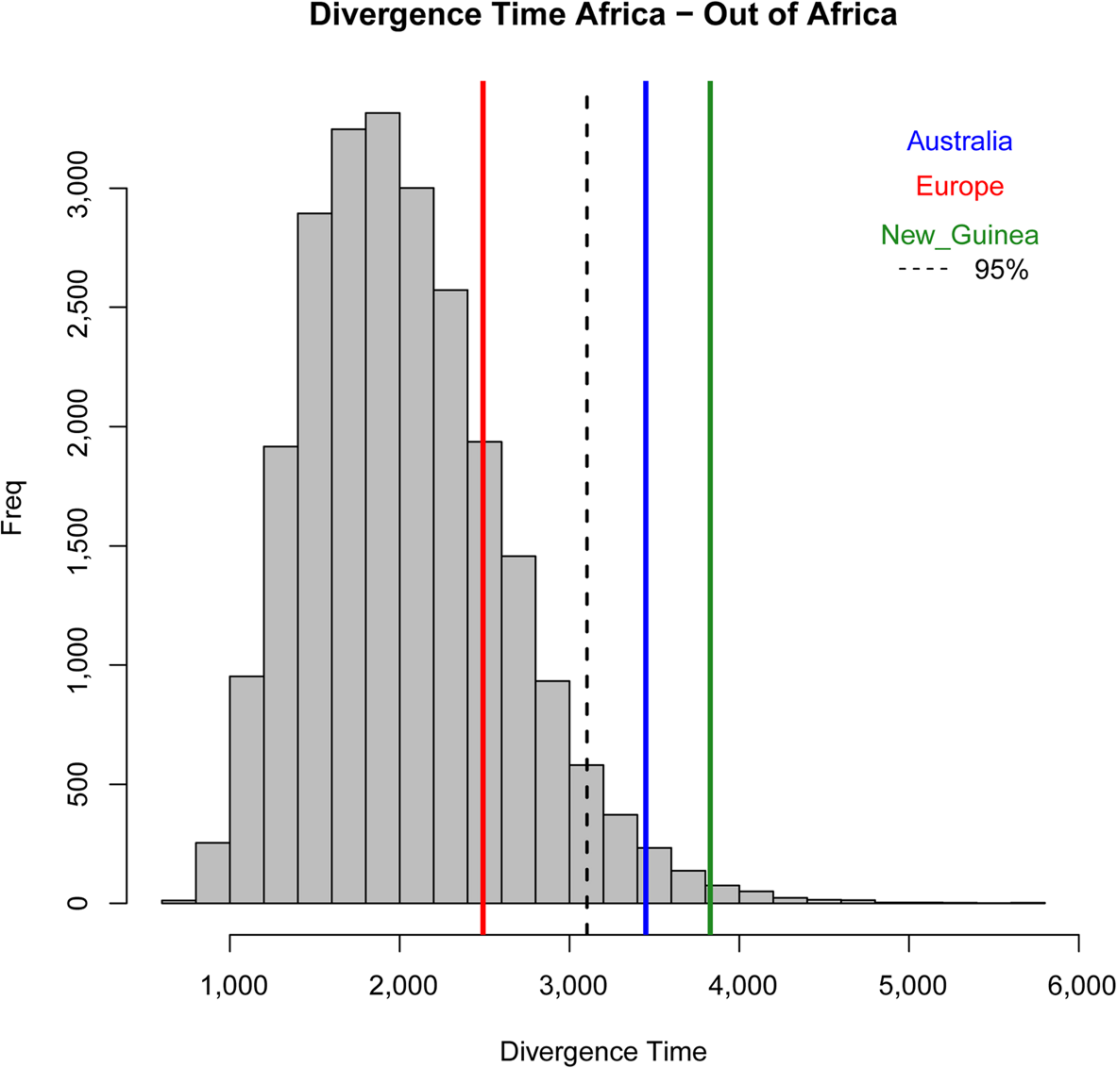
Comparison of three observed divergence times with the distribution of 24,000 divergence times between East African and non-African populations generated by simulation of a SD model. Data generated for 24 combinations of effective population sizes (3000 ≤ *N*_e_ ≤ 8000) and divergence times (40K years ago ≤ *T* ≤ 70K years ago), 1000 independent datasets for each such combination. At every iteration, genetic variation at 1 Mb was considered in 100 chromosomes per population, thus analyzing 200,000 Mb for each parameter combination (for a total of 4800 Gb in 24,000 iterations)

Having shown that significantly different times of separation from Africa are estimated for Europe and Australia/New Guinea, the question arises whether it would be possible to obtain such results by chance alone, had AMH dispersed in a single wave, at the time period at which that dispersal is generally placed (in the calculations that follow, we always considered the *N*_e_ and *T* estimates obtained using the unweighted *r*^2^ statistic). To answer, we needed a null distribution of *T* values under the SD model, which we constructed by simulation, using the software ms [56]. We plotted the (null) distribution of the 24,000 separation times derived from the simulations, and we compared it with the observed *T* estimates. Whereas the value estimated in the European sample falls perfectly within the range of times predicted by the classical SD model, that is not the case for the New Guinean and the Australian values, falling in the right tail of this distribution at *P* < 0.05 level (Fig. 4). This can only mean that a single exit from Africa, even considering the uncertainty in our knowledge of the relevant parameters, cannot account for the differences in the separation times from Africa observed, respectively, in Europe on the one hand and in Australo-Melanesia on the other. We obtained the same results also simulating three serial founder effects after the dispersal from Africa (see Additional file 3b), meaning that our results cannot be simply explained by accelerated drift in the Australo-Melanesians.

### Possible effects of a Denisovan admixture in Melanesia

Recent analyses of the genetic relationships between modern humans and Denisovans suggested that a fraction possibly as high as 6–8 % of the Melanesian genomes may be traced back to Denisovan ancestor [60]. To rule out the possibility that the apparent difference in African divergence times for Europe and Australo-Melanesia may somewhat reflect Denisovan admixture, we removed from the analysis the SNPs that were identified as representing the Denisovan contribution to the latter’s genome. We recalculated the *F*_ST_s from the 80,621 SNPs obtained from the filtering process and reestimated the divergence times from Africa, finding they are still very close to those previously estimated (Additional files 10 and 11).

### Possible effects of European back migration to Africa

Another realistic explanation for the observed pattern could also involve a simple isolation-by-distance model, in which geographically close populations are expected to be genetically close due to gene flow. Under this model, Europeans are expected to exchange more migrants with Africa than more remote populations of East and Southeast Asia, and Australo/Melanesia, which would bias downwards their divergence time estimates. To test for this possibility, we performed two different analyses, both estimating the extent of the European genetic contribution to the African gene pool considered in the present work. First, we ran a TreeMix [62] analysis using all the samples we considered (Additional file 12). This method estimates from genome-wide data a maximum likelihood tree of populations and then infers events of gene flow after the split by identifying populations that poorly fit the tree; if admixture was extensive, we expect to observe extensive reticulation in the tree. The calculated number of best-fitting migration events was five (Additional file 13), none of them involving exchanges between Europe and Africa. We also continued to add migrations up to 15 events, without finding any evidence of gene flow from Europe into Africa. Then, we moved to explicitly consider the admixture model embedded in the three-population test [34], calculating the *f*_3_ statistic. We considered one of the African samples as the target (East Africa, South Africa, West Africa) and other African samples and one non-African population as the sources of admixture. The results (Additional file 14; sheet named “target_Africa_source_Africa”) showed that, when considering East Africa as the target population, the comparisons involving Europe and Caucasus actually generate a significant *f*_3_ statistic, possibly indicating a genetic contribution in the East Africa sample from a European gene pool. However, significant *f*_3_ values have also been found in all the other comparisons, with significant admixture signals to East Africa from Melanesia, East Asia, and Central Asia. When the target sample is represented by West Africa or South Africa, we obtained no significant results. Taken together, these results may indicate that (a) if there has been any bias in the estimated divergence times between East Africa and Europe and between East Africa and Caucasus due to the back-to-Africa process, this bias should also affect in the same way all other divergence times estimate; (b) this bias, being shared among all samples analyzed, would affect the *punctual* estimated value of the divergence times and not the *differences* between estimates; and (c) the differences in the divergence times estimated for West Africa and South Africa, which are not showing any admixture signals with out-of-Africa populations, are credible.

### Estimates of population admixture in Southeast Asia

In our ADMIXTURE analysis, we identified an ancestral component (green, from *k* = 4) that is widely present in Australia and New Guinea and much less so in neighboring populations. To understand whether this component can actually be considered an ancestral marker of the first migration of modern humans out of Africa rather than the results of other population genetic phenomena (i.e., drift), we calculated the *f*_3_ statistic considering Australia or New Guinea as the target population (Additional file 14). We did not find evidence of admixture in any of the 506 comparisons we made; thus, it seems logical to consider this “green” component as representative of an ancient, geographically restricted gene pool (as also suggested by the divergence time estimates of these populations), even if its presence can also be related to a shared recent drift in these groups. Other Far Eastern populations, besides Australia and New Guineans for which we estimated a remote separation from Africans, may have taken part in an early exit from Africa through a Southern route. Identifying them is not straightforward, though, because we basically have a continuous set of divergence times from East Africa, from 66K to 107K years ago (Table 1). This result is consistent with both a continuous migration process from Africa across some 40K years (which so far has never been proposed, to the best of our knowledge) and an early exit, followed by genetic exchanges with later-dispersing groups, which has diluted or erased altogether the genetic evidence of the earliest migration.

After associating the Australo-Melanesian genotypes with the green ADMIXTURE component, we explored the possibility that the same component be a marker of the earliest African exit in other populations as well. To understand whether that could have actually been the case, we used TreeMix [62], selecting from our dataset just the populations showing at least 30 % of the green ADMIXTURE component at *k* = 5 and clustering together in the third group of the DAPC scatterplot. We chose the East Asia sample as outgroup. Additional file 15 shows the maximum likelihood tree. Evidence for genetic exchanges after population splits is apparent from East Asia (represented in light blue in the tree) toward populations putatively involved in the early African dispersal (represented in green in the tree). Prior to adding these migration episodes, the graph captures 87 % of the global variance in the data; including the top six migration events (indicated by arrows colored according to the intensities of the process) brought this percentage to 99 % (Additional file 16). Therefore, these results are consistent with the view that relatively recent admixture events have obscured the genomic signatures of the first migration out of Africa in these Southeast Asian populations, ultimately biasing downwards the estimates of their divergence times from Africans.

### Comparing the predictions of single vs multiple African exit models: geographical patterns

To conclude, we tried to better define some details of the AMH dispersal out of the African continent by evaluating which geographical migration route can better account for the current patterns of genome diversity. To minimize the effects of recent gene flow unrelated with the first human dispersals, which was clearly not negligible (see the previous section), we selected populations with at least 80 % of a single ancestral component in the ADMIXTURE results (i.e., Australia, Caucasus, East Africa, East Asia, Europe, New Guinea, South Africa, South India, West Africa). All the Mantel correlations thus calculated were positive and significant (Table 2), suggesting that all tested models succeed in plausibly predicting the observed patterns of genome diversity. The highest correlation observed for model 3 (*r* = 0.767) supports the Southern route hypothesis for populations of Southeast Asia and Oceania, but the difference between models 3 and 1 is not significant by Fisher’s criterion [68] (*Z* = −1.26, *P* = 0.08).

**Table 2:** Partial Mantel correlations between genetic and geographic distances

	Partial Mantel test	
	*r*	*P* value
Model 1	0.67	0.0001
Model 2	0.64	0.0012
Model 3	0.77	0.0001

Comparisons of the genetic distance matrix (*F*_ST_) with the geographic distances calculated according to the three dispersal models, while holding constant population divergence values (*T*). Values are Pearson correlation coefficients, and the *P* values have been empirically calculated over 10,000 permutations of one matrix’ rows and columns

## Discussion

Establishing whether genomic differences among populations are compatible with a single major expansion from Africa is crucial for reconstructing the set of migration processes leading to the human peopling of the Old World. Two difficult-to-disentangle factors, namely the effects of population sizes and of admixture after the main population split, complicate the exercise. Past population sizes are unknown, and are generally estimated from genetic diversity, under neutrality assumptions. However, genetic differences between populations are large if the populations long evolved independently, or if they had small effective sizes, or by any combination of these factors. To circumvent this problem, in this study we resorted to LD values to estimate long-term population sizes and separate their effect from that of population history. This way, we found that the populations at the extremes of the geographical range considered differ substantially in the timing of their separation from the East African populations. This difference is statistically significant, and we showed by simulation that it cannot possibly be reconciled with a model assuming a single major dispersal of all non-Africans (whether or not followed by a series of founder effects) through the classical northern route (as pointed by Pagani et al. [69]). The model we tested is necessarily simple and does not take into account potential admixture with archaic human forms. However, since the estimated degree of Neandertal ancestry is the same in all modern non-African populations [4], the inclusion of this event would only affect the *absolute* values of divergence times from Africa and not the *ratio* between them.

Conversely, two processes may actually have a potentially more serious confounding effect on these calculations; Denisovan admixture may inflate the estimated divergence times in the easternmost populations, and back migration from Europe into Africa may have the opposite effect upon the European populations. Removal of the SNPs identified as a potential Denisovan contribution to the modern genomes caused no substantial change in the results, and the analysis of *f*_3_ statistics did not show any detectable impact of back migration to Africa upon the set of markers we considered. To exclude the possibility that a higher level of African ancestry in the European samples we considered (perhaps due to back migration into Africa from Southern Europe) is responsible for the observed pattern, we ran additional comparisons of divergence time distributions between sub-Saharan Africans and Europeans with different geographic origins. To do this, we analyzed Basque and English populations from the POPRES dataset [70] and the Finns from the 1000 Genomes Project [71]. The divergence times estimated for these populations are very close to those previously estimated for Europeans (data not shown); therefore, it does not seem that the presumably higher level of African admixture in southern than Northern Europeans may possibly account for our results. These findings show that our estimates reflect to a minimal extent, if any, the effects of potentially confounding factors related with interbreeding with Denisovans [60] or gene flow with other modern humans, after the African expansions.

As for admixture among modern populations after the split from Africa, which may certainly affect estimates of their divergence time [72], the demographic history of large sections of Asia is too complex and elusive to be addressed in a general study, such as this, without detailed modeling of each local process. However, we argue that the impact of modern admixture upon our results cannot be too strong, because geographically intermediate populations were excluded from the final analysis. This way, significant differences in time estimates were observed for populations (Europe, Caucasus, New Guinea, and Australia) showing a rather homogeneous genetic composition in the ADMIXTURE analysis, with most individual genotypes attributed to a single ancestral component (Fig. 3).

The method used in the present study allows us to safely rule out that fluctuations in long-term population sizes might have distorted our time estimates. Threefold differences in very ancient (e.g., >100,000 years ago; Additional file 7) population sizes may appear, at first sight, difficult to justify, because at that time all *N*_e_ values should converge to a value representing the size of the common ancestral African population. However, a similar result was also obtained in the only previous study based on the same method [18] and interpreted as reflecting founder effects accompanying the dispersal from Africa. In turn, these phases of increased genetic drift may have increased LD and hence caused underestimation of *N*_e_ in all non-Africans. However, the resulting distortion, if any, should have affected the *absolute* values of *T*, but not the *relative* timing of the Europeans’ and Asians’ separation from Africans, which is what this study is concerned with. Another possibility is that 100,000 or so years ago, the ancestors of current Eurasians were already genetically distinct from the ancestors of modern Africans (as proposed by [73, 74]). If so, the different *N*_e_ estimates of the present study would not be a statistical artifact but would reflect actual differences between geographically isolated ancient populations. Incidentally, we note that McEvoy et al. [18] estimated very recent dates of separation from the African population (36,000 years ago for Europe, 44,000 years ago for East Asia). In the present study, based on a wider geographical coverage, we obtained older dates, better compatible with archaeological estimates [75].

Two independent analyses (by ADMIXTURE and TreeMix) suggest that the genotypes of most Central Asians reflect variable degrees of gene flow between populations which may have left Africa in different waves. As a result, the distribution of divergence times is essentially continuous, and hence, it would make no sense to try to classify Central Asian populations as derived from either the first or the second African exit under the model of multiple dispersals.

When we modeled population dispersal in space, the correlation between genetic and geographic distances was higher under the MD than under the SD model, but this difference was statistically insignificant (Table 2). This seems likely due to the fact that the three models being compared share several features, such as the same set of geographic/genetic distances for the European populations, which reduces the power of any test. However, the separation times previously estimated made us confident that the SD model is inconsistent with the data, and so what was really important at this stage was the comparison between the two MD models. The better fit of model 3 than of model 2 implies that the MD model works better if only part of the Asian genomic diversity is attributed to the earliest dispersal. A better fit of a MD than of a SD model was also observed in parallel analyses of cranial measures and of a much smaller genomic dataset [13], suggesting that our findings may indeed reflect a general pattern of human diversity.

The data we analyzed are probably affected, to an unknown but not negligible extent, by a bias due to the fact that most SNPs in the genotyping platforms were discovered in European populations; however, the approach we used to calculate *N*_e_ and hence the separation time, through LD, is expected to be relatively unaffected by this kind of bias [18, 76]. At any rate, a likely effect of such a bias would be a spurious increase of the estimated differences between Europeans and the populations being compared with them, Africans in this case. Quite to the contrary, here, the Europeans appeared significantly *closer* to Africans than Australo-Melanesians, a result which therefore cannot be due to that kind of ascertainment bias. Moreover, our *F*_ST_ estimates are comparable with those obtained by the last survey of the 1000 Genomes Project; when rare variants are excluded (MAF > 0.05) and for the populations of this study, the average *F*_ST_s differ by less than 1 %. This means that we are not losing power considering a subset of SNPs and that the ascertainment bias did not substantially affect our estimates.

**Fig. 5:**
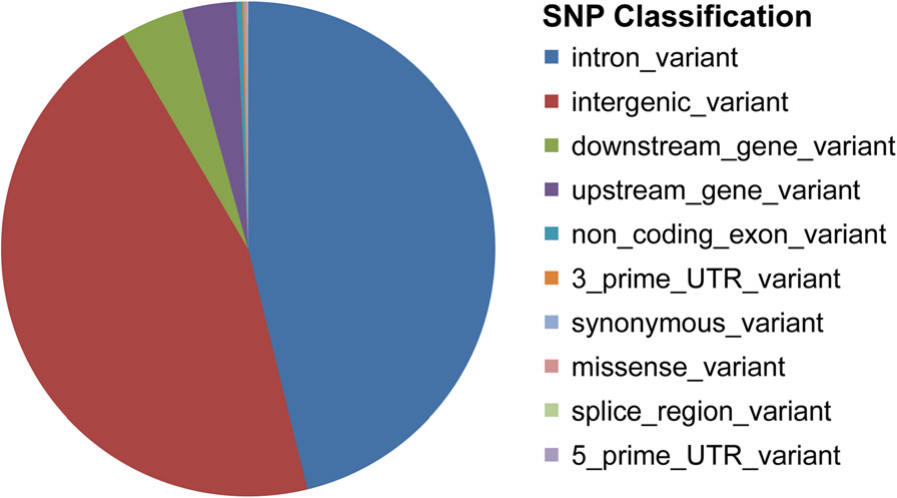
Distribution of the SNPs considered in functionally different genome regions

Can selection account, at least in part, for these findings? In principle, we have no way to rule this out. However, in practice, even though positive selection may have extensively affected the human genome, large allele frequency shifts at individual loci are surprisingly rare [77], so much so that so far only for very few SNPs any effects of selection have been demonstrated [78]. If we also consider that genomic regions with large allele frequency differences are not generally associated with high levels of LD, in contrast with what would be expected after a selective sweep [77, 79], it seems fair to conclude that the main allele frequency shifts occurred in a rather remote past and are unlikely to reflect geographic differences in the selection regimes [80]. In any case, only 8 % of the SNPs were considered map within expressed loci, or in their control regions (Fig. 5); therefore, the impact of selection upon the results of this study, if any, can hardly be regarded as substantial.

## Conclusions

Analyses of genomic data based on different sets of assumptions and different methods agree in indicating that (i) a model with a single early dispersal from Africa fails to account for one crucial aspect of human genome diversity, the distribution of divergence times from Africa, and (ii) within the model of multiple dispersal, geographical patterns of genome diversity are more accurately predicted assuming that not all Asian and New Guinean/Australian populations have had the same evolutionary history (question 1 of the “Background” section). Conversely, we could not show a significant difference in the fit of the geographical models we tested; although we can confidently rule out the hypothesis of a single dispersal, establishing that the exact migrational routes from Africa into Asia will require further efforts (question 2 of the “Background” section).

In the light of these results, we propose that at least two major dispersal phenomena from Africa led to the peopling of Eurasia and Australo-Melanesia. These phenomena seem clearly distinct both in their timing and in their geographical scope.

The view whereby only part of the ancestors of current non-African populations dispersed through the Levant has some non-trivial consequences upon the possible interactions between AMH and archaic forms, traces of whose genomes have been identified in many non-African populations, including New Guineans [4, 81]. The estimated contribution of Neandertals is less in the European than in the Asian/Melanesian genomes, despite the long coexistence between Neandertals and Europeans [82]. At present, the standard way to explain this finding is to assume one single, major episode of hybridization in Palestine (or perhaps further north and east [83]) 47K to 65K years ago [58], followed by a split between the Europeans’ ancestors on the one hand and the Asians’ and Oceanians’ on the other [83, 84]. After that, additional contacts might have occurred but only between Neandertals and Asians [85]. However, if most ancestors of New Guineans dispersed through a Southern route, as this study shows, they would have missed by 2000 km or so the nearest documented Neandertals with whom they could have intercrossed. Thus, this study raises the possibility that the current patterns of human diversity need more complex models to be fully explained. One possibility is that admixture with Neandertals might have occurred before AMH left Africa [86]. Another is that common ancestry, rather than hybridization, may account for the excess similarity of Eurasians with Neandertals, in the presence of an ancient structuring of populations [74, 87]. A third possibility is that the apparent traces of Neandertal hybridization in Papua New Guinea may in fact be due to Denisovan admixture. We are not in a position to actually test for these possibilities, but exploring these hypotheses may contribute to a better understanding of the main human dispersal processes and of the relationships between archaic and modern human forms.

## Additional files

Additional file 1:
**Geographic location of all the 71 populations analyzed.** The different datasets we use are represented by different colors and are detailed in Additional file 2. (TIFF 915 kb) Additional file 2:
**General information about the 24 metapopulations analyzed.** (PDF 342 kb) Additional file 3:
**Representation of the human demographic models tested by ms.** The past is at the top, the present is at the bottom; (a) single founder effect; (b) serial founder effects. (PDF 181 kb) Additional file 4:
**Estimation of the most likely number of clusters in the data (** ***X*** **-axis) as a function of the cross-validation error observed in the attempted assignments (** ***Y*** **-axis).** (PDF 84 kb) Additional file 5:
**Inference of the most likely number of clusters in the DAPC.** A *k* value of 6 (the lowest BIC value) represents the best summary of the data. (PDF 190 kb) Additional file 6:
**Discriminant Analysis of Principal Components, DAPC.** (a) Classification of individual genotypes; for each row (each population), the figures refer to the numbers of individuals assigned to the *k* = 6 clusters, each cluster associated with a different color; (b) scatterplot along the first two axes; each symbol corresponds to an individual genotype; in the insets, the fraction of principal components retained in the analysis (left) and the fraction of the overall variance attributed to the first five eigenvalues, with the first two columns, in gray, representing the first two discriminant functions (right). (PDF 1284 kb) Additional file 7:
**Estimates of** ***N***_**e**_ **from measures of linkage disequilibrium, using the (a)** ***r*** ^**2**^ **and (b)** ***σ*** ^**2**^ **statistics as estimators of LD level.** Time is on the *X*-axis and is expressed in generations from the present. Very recent estimates have been omitted because they were not reliably estimated. (PDF 185 kb) Additional file 8:
**Harmonic means of the estimated population effective sizes (** ***N***_**e**_ **), using the (a)** ***r*** ^**2**^ **and (b)** ***σ*** ^**2**^ **statistics as estimators of LD level.** Vertical bars represent empirical 95 % confidence estimates. (PDF 165 kb) Additional file 9:
**Pairwise** ***F***_**ST**_ **values estimated between populations.** The matrix is symmetrical. (PDF 761 kb) Additional file 10:
**Estimates of population divergence times from East, West, and South Africa.** (PDF 193 kb) Additional file 11:
**Population divergence time estimated on a subset of SNPs chosen to exclude the effect of an archaic introgression from Denisovan.** (PDF 187 kb) Additional file 12:
**Population relationships inferred by TreeMix.** The maximum likelihood tree is in black; branch lengths are proportional to the impact of genetic drift, which may or may not faithfully represent separation times between populations. The inferred migration events are represented by arrows pointing from the putative source to the putative target populations, with colors of the arrows representing the relative weight of the genetic exchanges, according to the heat scale on the left. (PDF 112 kb) Additional file 13:
**Fractions of the total variance explained by the model at increasing numbers of migrations superimposed to the bifurcating tree in the TreeMix analysis.** (PDF 92 kb) Additional file 14:
***f***_**3**_ **statistic results.** (XLS 81 kb) Additional file 15:
**Population relationships inferred by TreeMix, considering just the populations showing at least 30 % of the green ADMIXTURE component at** ***k*** **= 5 and clustering together in the third group of the DAPC scatterplot.** (PDF 131 kb) Additional file 16:
**Fractions of the total variance explained by the model at increasing numbers of migrations superimposed to the bifurcating tree in the TreeMix analysis, considering just the populations showing at least 30 % of the green ADMIXTURE component at** ***k*** **= 5 and clustering together in the third group of the DAPC scatterplot.** (PDF 89 kb)

## Abbreviations

AMH: anatomically modern humans
BIC: Bayesian information criterion
cM: CentiMorgan
DAPC: Discriminant Analysis of Principal Components
IBD: identity by descent
LD: linkage disequilibrium
MAF: minor allele frequency
MD: multiple dispersals
PC: principal component
rv: resistance value
SD: single dispersal
SNP: single nucleotide polymorphism
