# Supplementary figures and images for: Early modern human dispersal from Africa: genomic evidence for multiple waves of migration
Source: Investig Genet. 2015 Nov 6;6:13. doi: 10.1186/s13323-015-0030-2 (PMC4636834; doi:10.1186/s13323-015-0030-2)

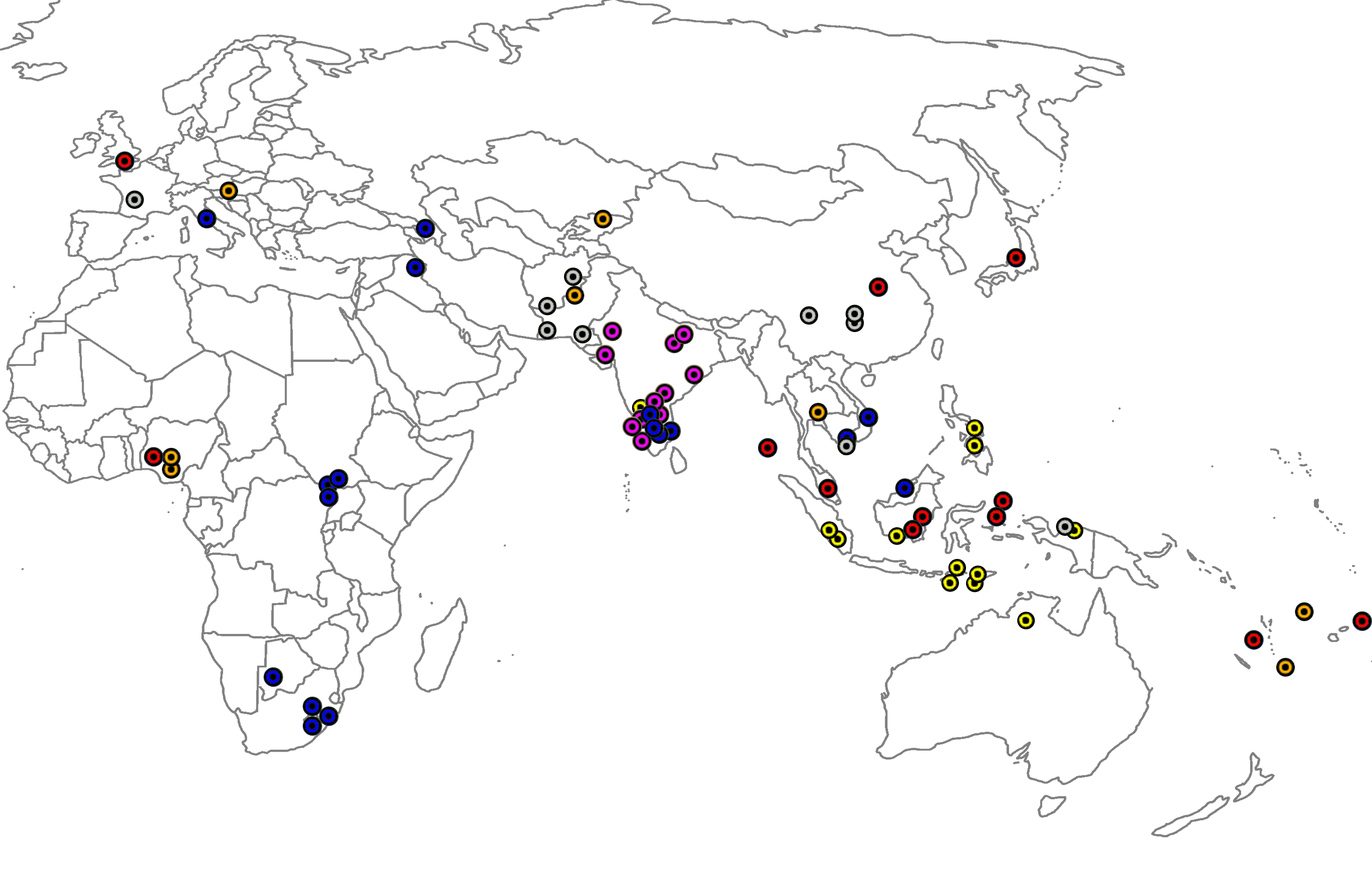

Supplement: Additional file 1: — Geographic location of all the 71 populations analyzed. The different datasets we use are represented by different colors and are detailed in Additional file 2. (TIFF 915 kb) [file 13323_2015_30_MOESM1_ESM.tif]

a

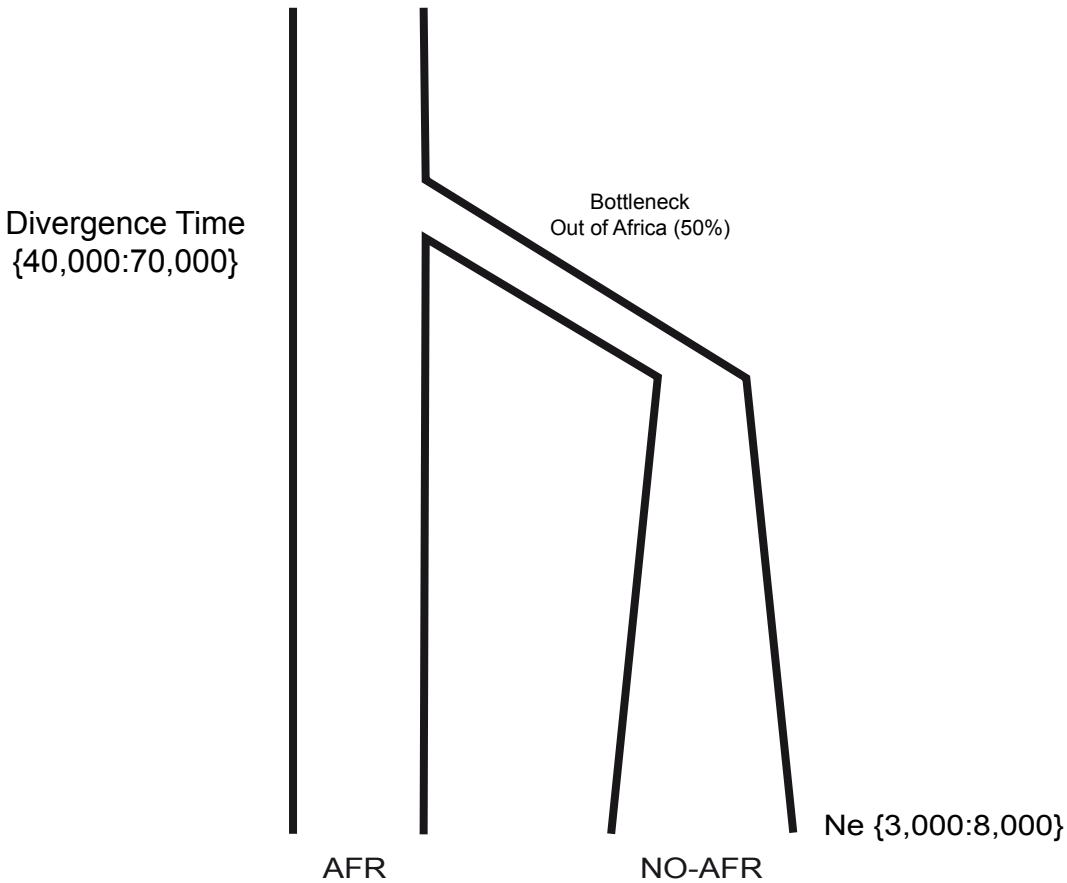

b

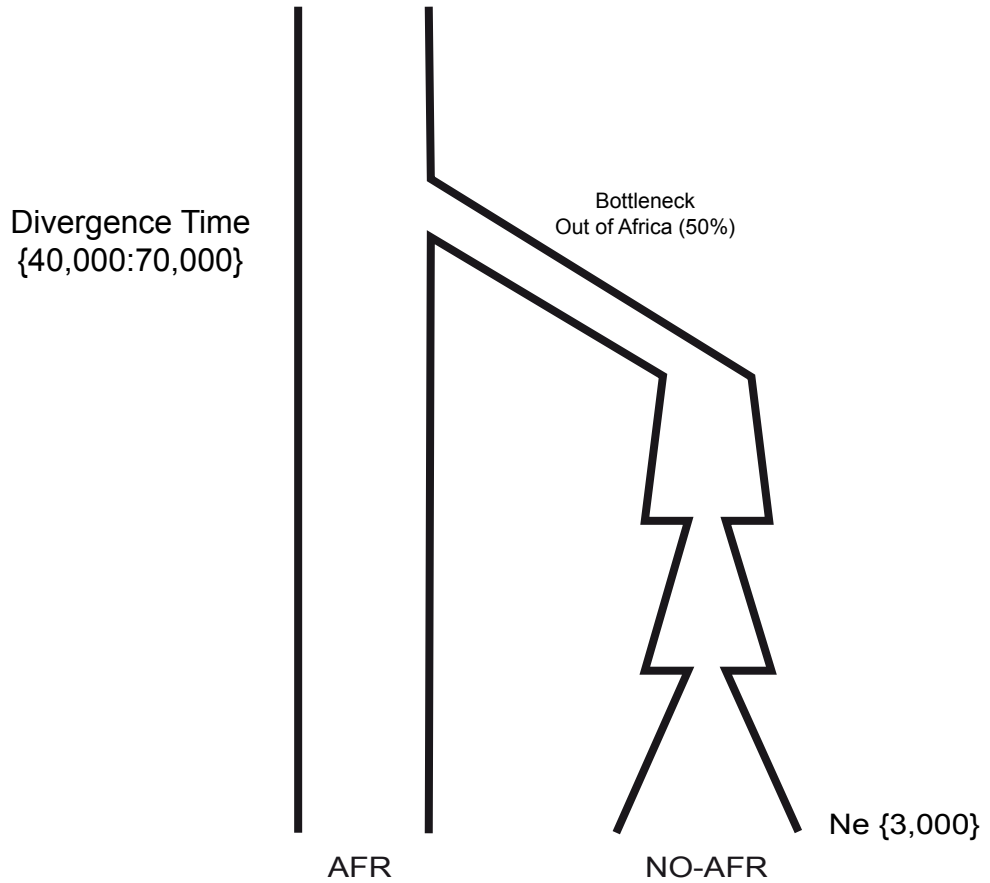

Supplement: Additional file 3: — Representation of the human demographic models tested by ms. The past is at the top, the present is at the bottom; (a) single founder effect; (b) serial founder effects. (PDF 181 kb) [file 13323_2015_30_MOESM3_ESM.pdf]

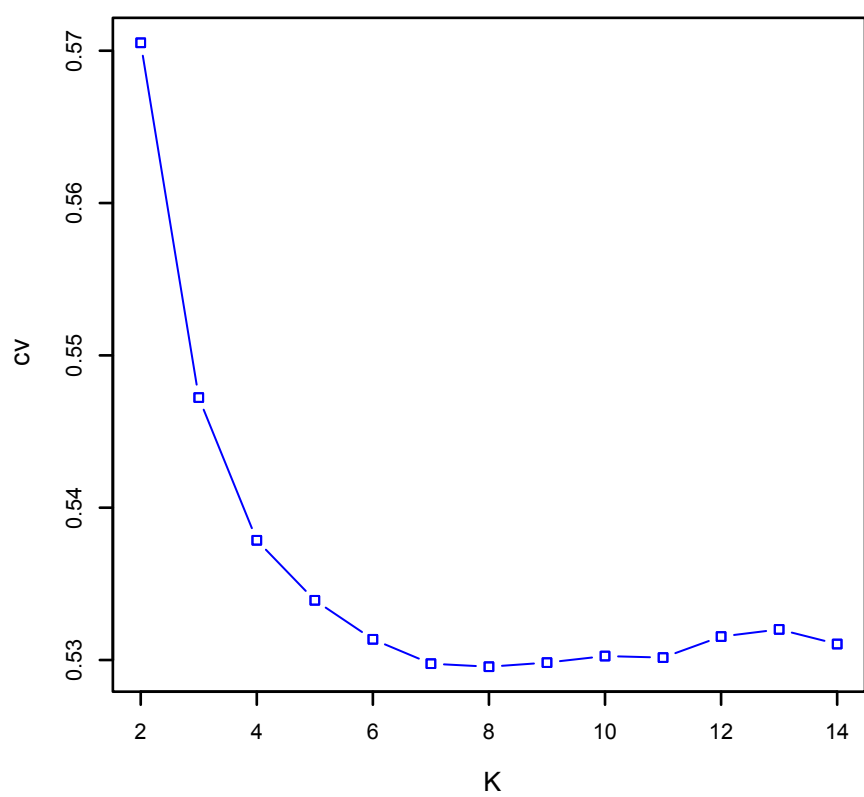

Supplement: Additional file 4: — Estimation of the most likely number of clusters in the data ( X -axis) as a function of the cross-validation error observed in the attempted assignments ( Y -axis). (PDF 84 kb) [file 13323_2015_30_MOESM4_ESM.pdf]

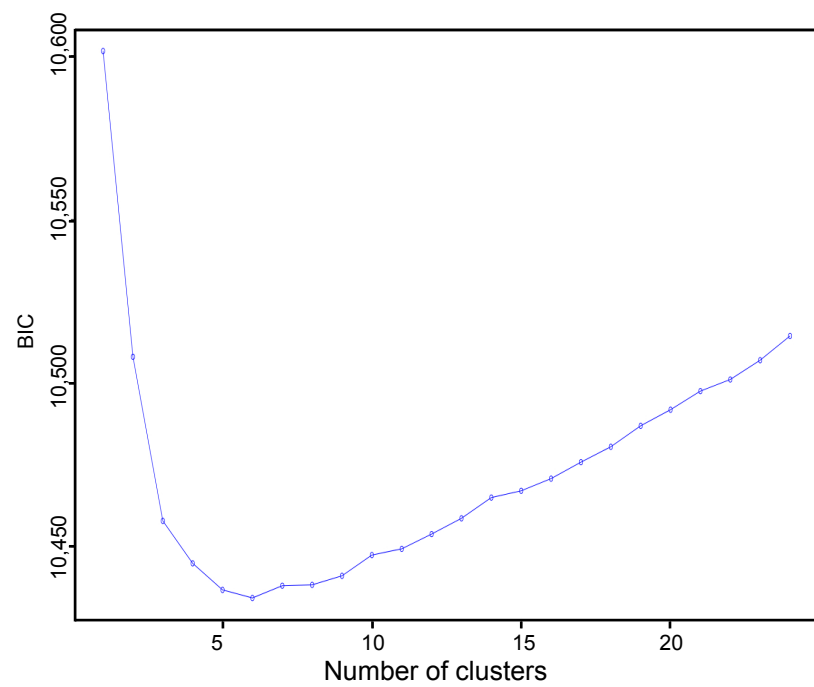

Supplement: Additional file 5: — Inference of the most likely number of clusters in the DAPC. A k value of 6 (the lowest BIC value) represents the best summary of the data. (PDF 190 kb) [file 13323_2015_30_MOESM5_ESM.pdf]

a

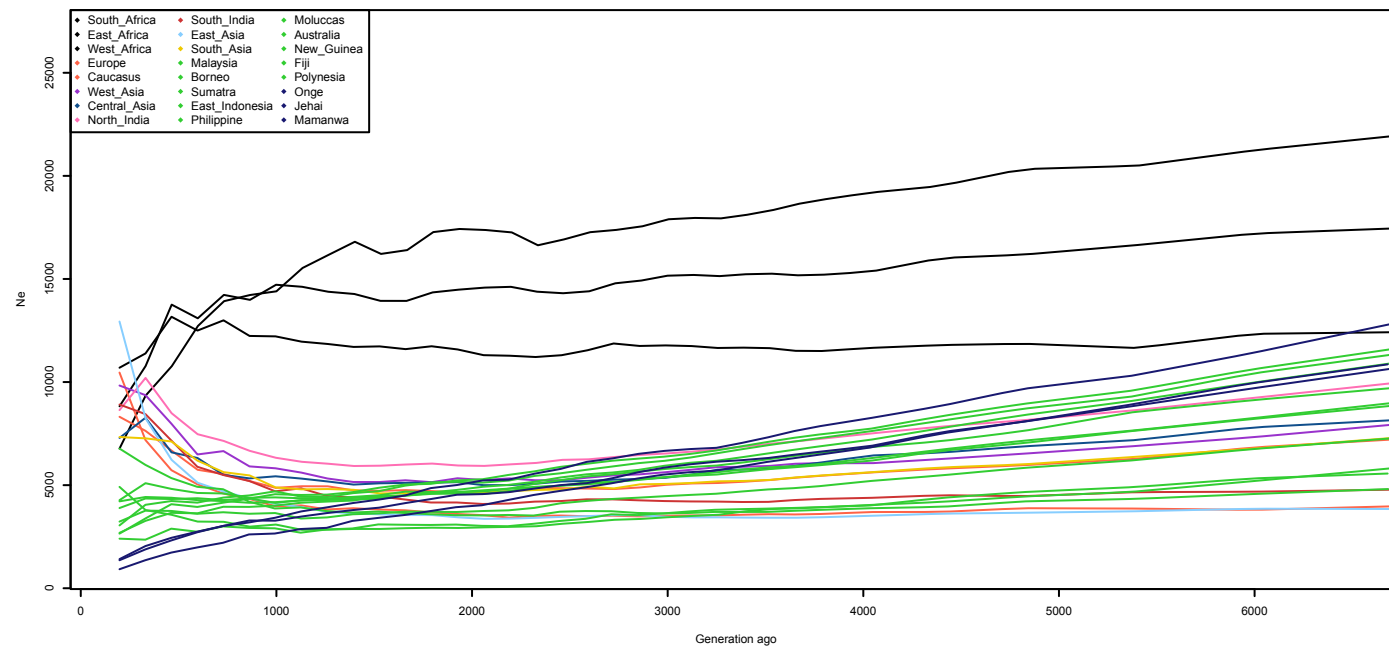

b

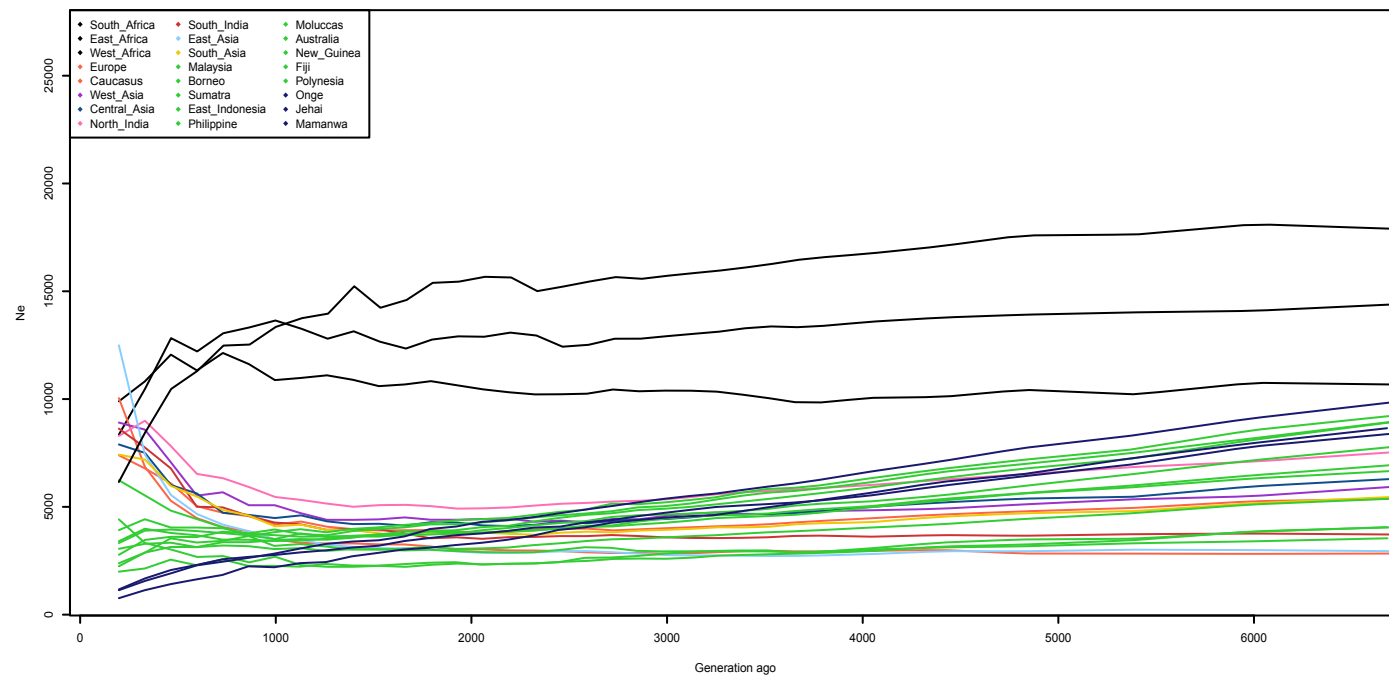

Supplement: Additional file 7: — Estimates of N e from measures of linkage disequilibrium, using the (a) r 2 and (b) σ 2 statistics as estimators of LD level. Time is on the X-axis and is expressed in generations from the present. Very recent estimates have been omitted because they were not reliably estimated. (PDF 185 kb) [file 13323_2015_30_MOESM7_ESM.pdf]

a

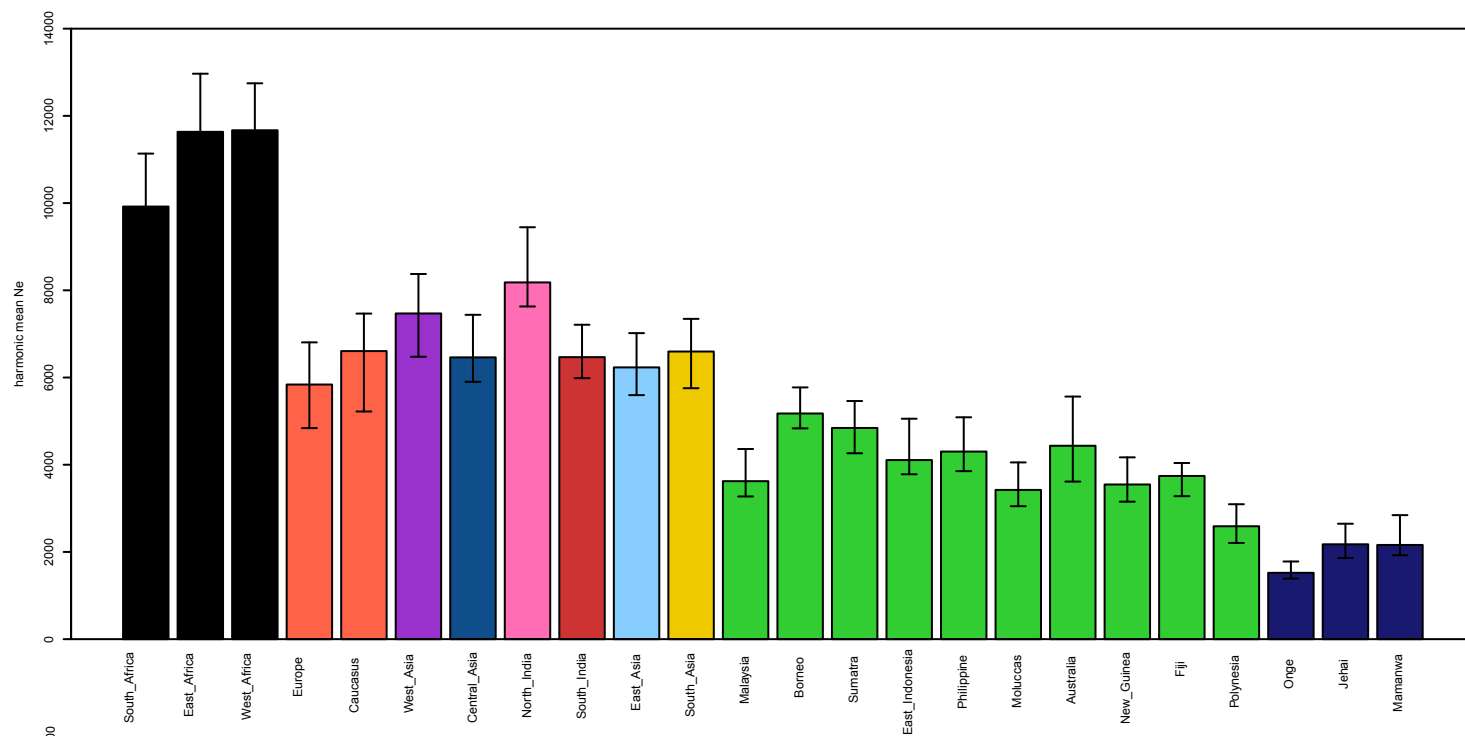

b

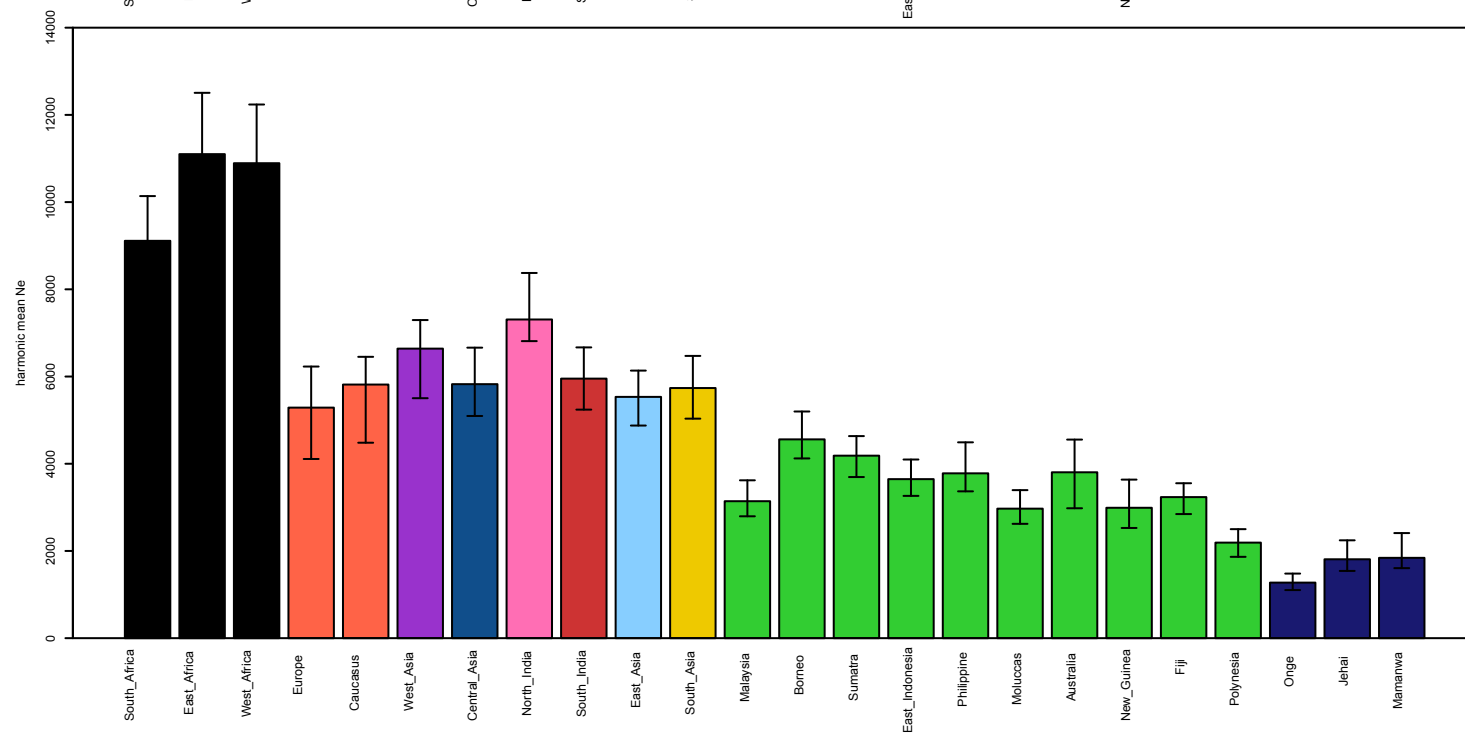

Supplement: Additional file 8: — Harmonic means of the estimated population effective sizes ( N e ), using the (a) r 2 and (b) σ 2 statistics as estimators of LD level. Vertical bars represent empirical 95 % confidence estimates. (PDF 165 kb) [file 13323_2015_30_MOESM8_ESM.pdf]

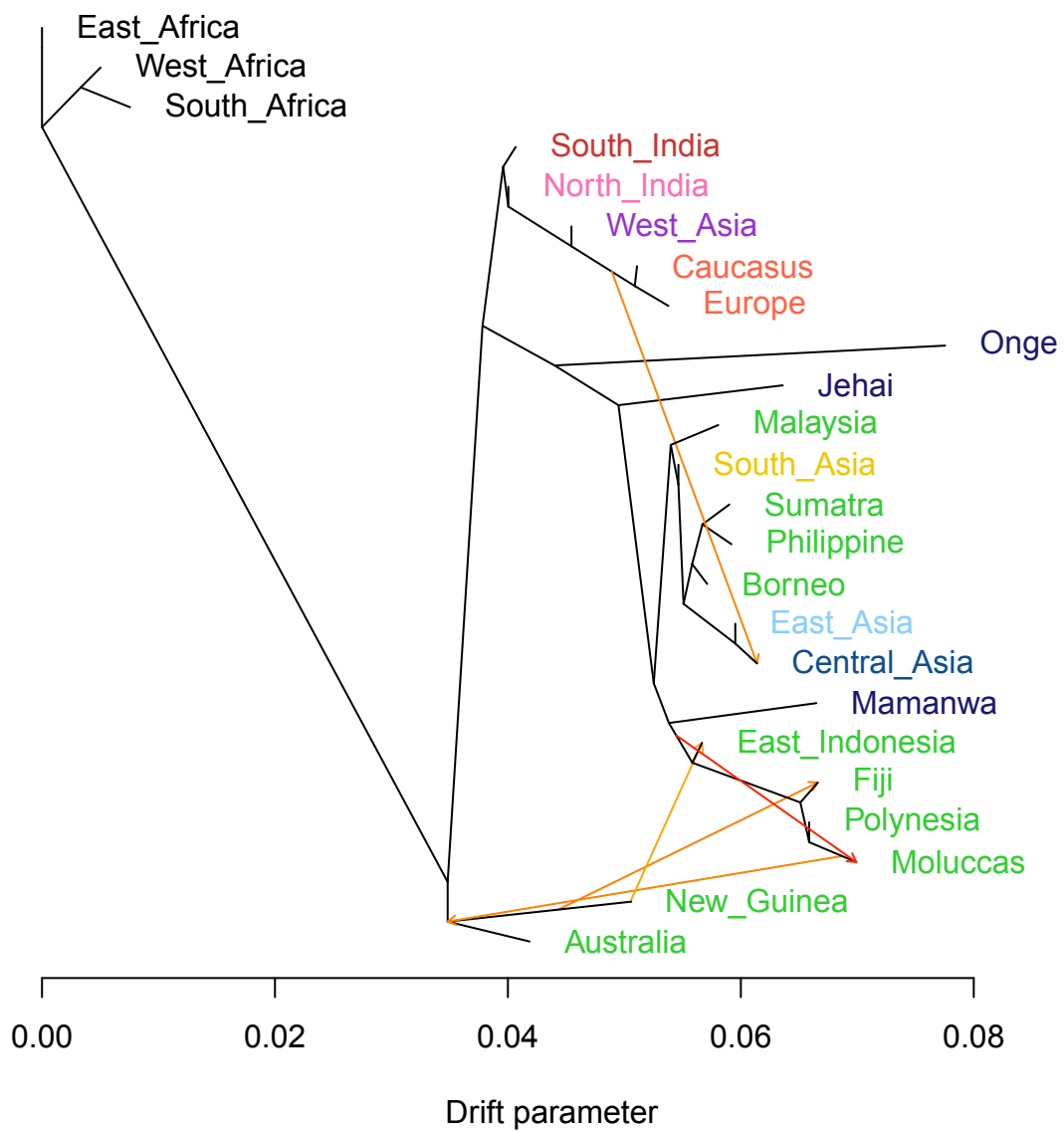

Supplement: Additional file 12: — Population relationships inferred by TreeMix. The maximum likelihood tree is in black; branch lengths are proportional to the impact of genetic drift, which may or may not faithfully represent separation times between populations. The inferred migration events are represented by arrows pointing from the putative source to the putative target populations, with colors of the arrows representing the relative weight of the genetic exchanges, according to the heat scale on the left. (PDF 112 kb) [file 13323_2015_30_MOESM12_ESM.pdf]

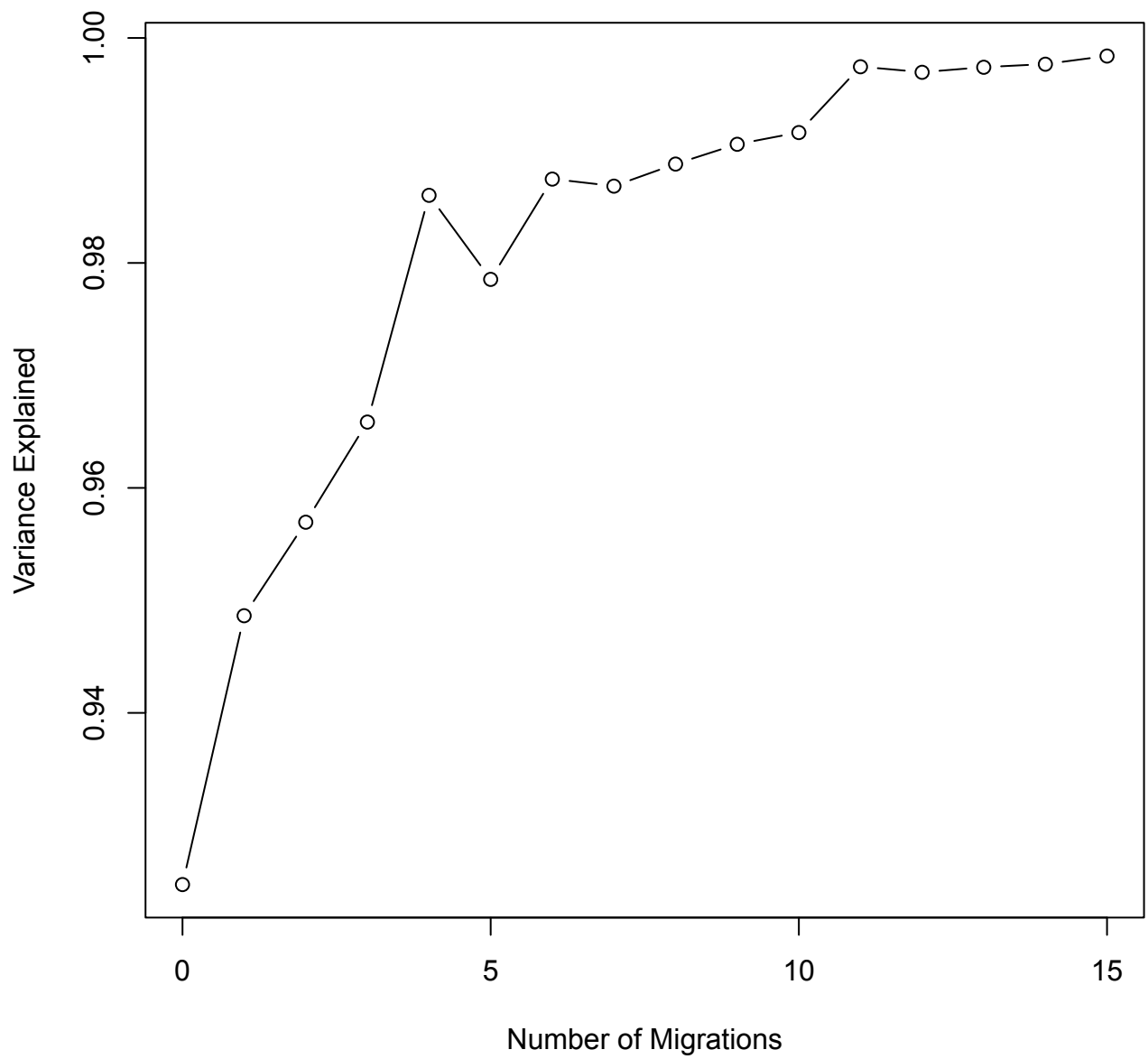

Supplement: Additional file 13: — Fractions of the total variance explained by the model at increasing numbers of migrations superimposed to the bifurcating tree in the TreeMix analysis. (PDF 92 kb) [file 13323_2015_30_MOESM13_ESM.pdf]

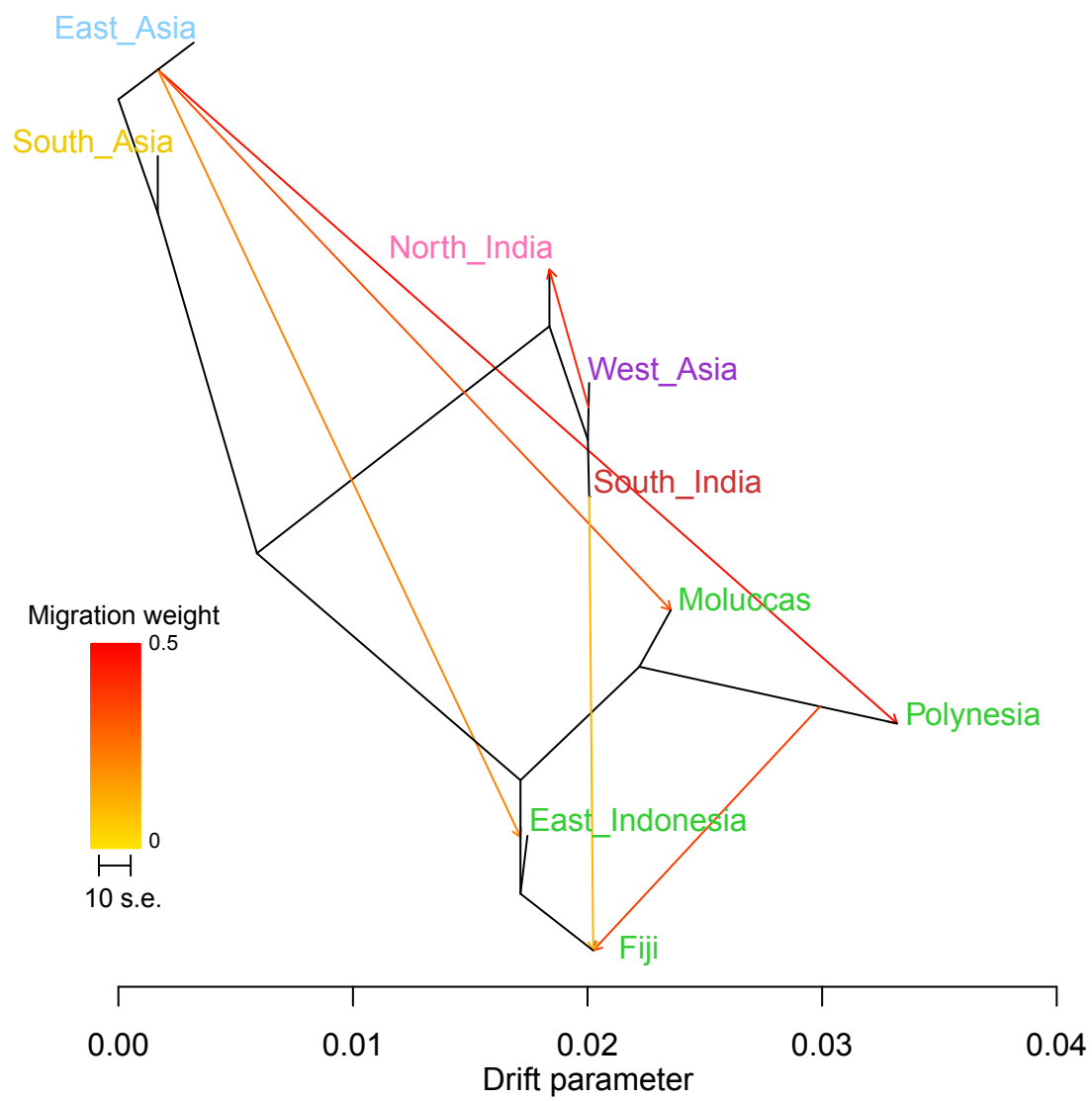

Supplement: Additional file 15: — Population relationships inferred by TreeMix, considering just the populations showing at least 30 % of the green ADMIXTURE component at k = 5 and clustering together in the third group of the DAPC scatterplot. (PDF 131 kb) [file 13323_2015_30_MOESM15_ESM.pdf]

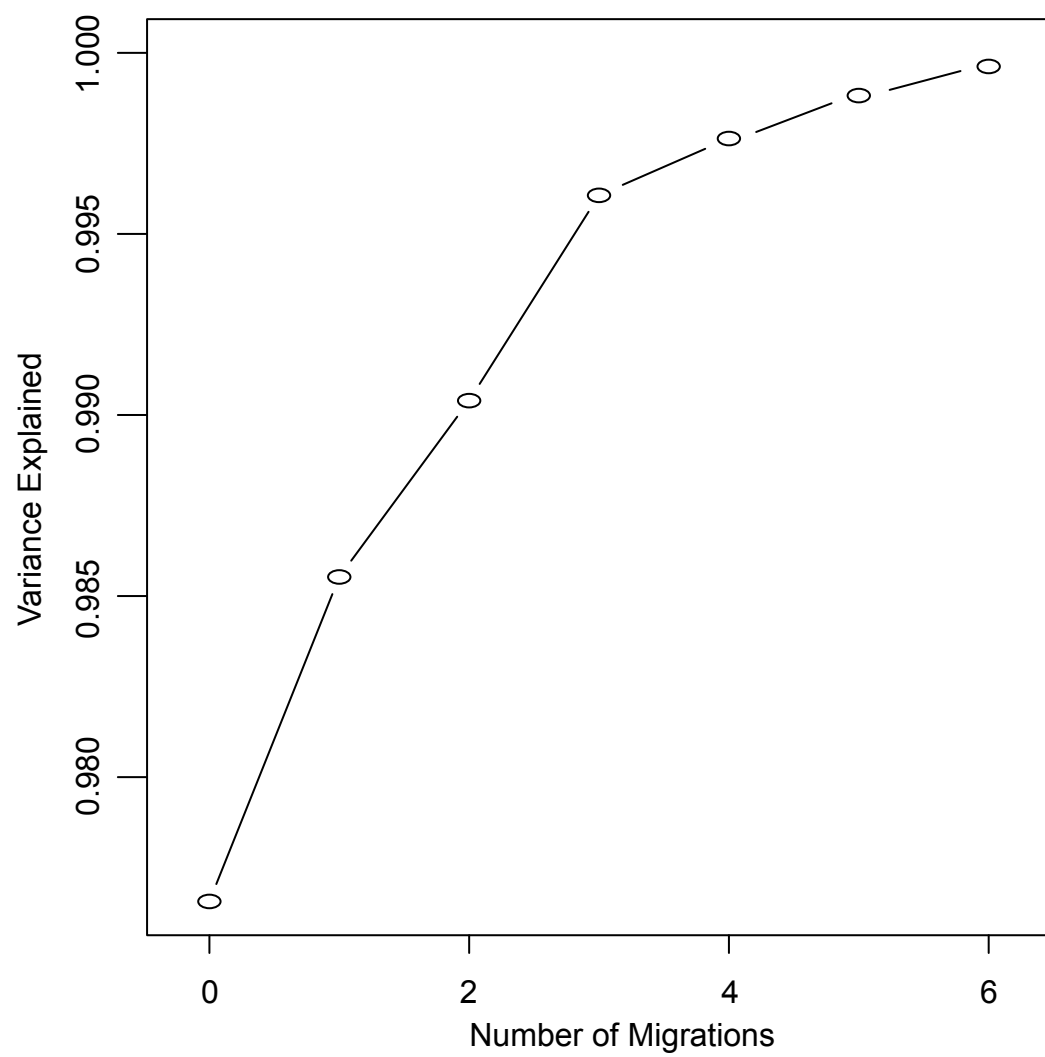

Supplement: Additional file 16: — Fractions of the total variance explained by the model at increasing numbers of migrations superimposed to the bifurcating tree in the TreeMix analysis, considering just the populations showing at least 30 % of the green ADMIXTURE component at k = 5 and clustering together in the third group of the DAPC scatterplot. (PDF 89 kb) [file 13323_2015_30_MOESM16_ESM.pdf]
