# Supplementary material for: Early modern human dispersal from Africa: genomic evidence for multiple waves of migration
Source: Investig Genet. 2015 Nov 6;6:13. doi: 10.1186/s13323-015-0030-2 (PMC4636834; doi:10.1186/s13323-015-0030-2)
Supplement: Additional file 2: — General information about the 24 metapopulations analyzed. (PDF 342 kb) [file 13323_2015_30_MOESM2_ESM.pdf]

Group	Population	Sample_Size	Reference
South Africa(37)	Pedi	9	35
	Nguni	9	35
	Sotho/Tswana	7	35
	!Kung	12	35
East Africa(46)	Luhya	22	35
	Alur	10	35
	Hema	14	35
West Africa(160)	Yoruba	111	5
	Dogon	24	36
	Bambara	25	36
Europe(166)	CEU	111	5
	Tuscan	25	35
	French	5	32
	Slovenian	25	36
Caucasus(42)	Urkarah	18	35
	Kurd	24	35
West Asia(38)	Pakistani	23	36
	Balochi	2	32
	Makrani	4	32
	Sindhi	4	32
	Pathan	5	32
Central Asia(23)	Kyrgyzstani	23	36
North India(25)	Bhil	7	34
	Meghawal	5	34
	Sahariya	4	34
	Satnami	4	34
	Lodi	5	34
South India(98)	Dravidian	11	33
	Chenchu	6	34
	Kurumba	9	34
	Hallaki	6	34
	Kamsali	3	34
	Madiga	4	34
	Mala	3	34
	T.N. Dalit	13	35
	Irula	22	35
	A.P. Mala	11	35
	A.P. Madiga	10	35
East Asia(188)	Japan	86	5
	Han Chinese	88	5
	Miaozu	5	32
	Tujia	5	32
	Yizu	4	32
South Asia(35)	Khmer	5	35
	Thai	20	36
	Cambodian	3	32
	Vietnamese	7	35
Malaysia(10)	Temuan	10	5
Borneo(73)	Land Dayak	15	33
	Barito River	23	5
	Bidayuh	10	5
	Iban	25	35
Sumatra(20)	Besemah	10	33
	Semede	10	33
East indonesia(10)	Flores	1	33
	Roti	4	33
	Timor	3	33
	Alor	2	33
Philippine(16)	Manobo	16	33
Moluccas(10)	Hiri	7	5
	Ternate	3	5
Australian(10)		10	33
New Guinea(27)	Papua New Guinea	24	33
	Papuan	3	32
Fiji(24)		24	33
Polinesia(44)	Polynesia	19	5
	Samoan	13	36
	Tongan	12	36
Onge(9)	Negrito	9	5
Jehai(8)	Negrito	8	5
Mamanwa(11)	Negrito	11	33
