# Supplementary material for: Early modern human dispersal from Africa: genomic evidence for multiple waves of migration
Source: Investig Genet. 2015 Nov 6;6:13. doi: 10.1186/s13323-015-0030-2 (PMC4636834; doi:10.1186/s13323-015-0030-2)

a

	1	2	3	4	5	6
Australia	0	0	0	10	0	0
Borneo	0	73	0	0	0	0
Caucasus	0	0	0	0	42	0
Central_Asia	0	23	0	0	0	0
East_Africa	46	0	0	0	0	0
East_Asia	0	188	0	0	0	0
East_Indonesia	0	3	7	0	0	0
Europe	0	0	0	0	166	0
Fiji	0	0	24	0	0	0
Jehai	0	8	0	0	0	0
Malaysia	0	10	0	0	0	0
Mamanwa	0	11	0	0	0	0
Moluccas	0	0	10	0	0	0
New_Guinea	0	0	0	27	0	0
North_India	0	0	0	0	0	25
Onge	0	0	0	0	0	9
Philippine	0	16	0	0	0	0
Polynesia	0	0	44	0	0	0
South_Africa	37	0	0	0	0	0
South_Asia	0	35	0	0	0	0
South_India	0	0	0	0	0	98
Sumatra	0	20	0	0	0	0
West_Africa	160	0	0	0	0	0
West_Asia	0	0	0	0	8	30

b

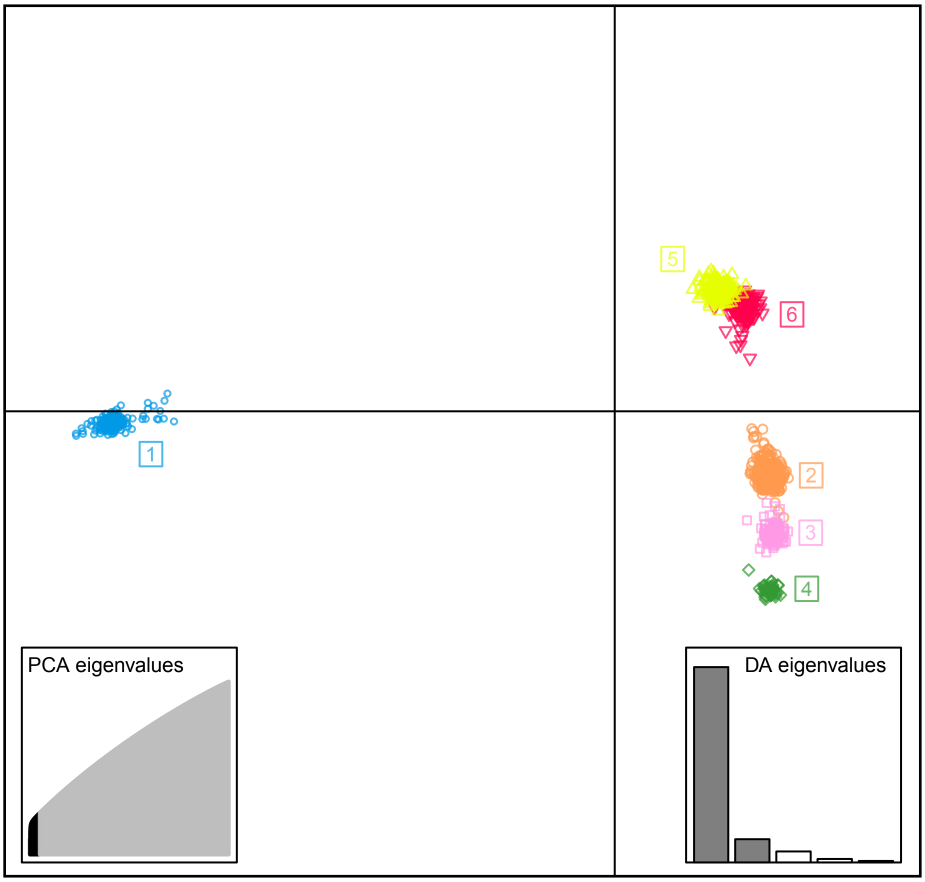

Supplement: Additional file 6: — Discriminant Analysis of Principal Components, DAPC. (a) Classification of individual genotypes; for each row (each population), the figures refer to the numbers of individuals assigned to the k = 6 clusters, each cluster associated with a different color; (b) scatterplot along the first two axes; each symbol corresponds to an individual genotype; in the insets, the fraction of principal components retained in the analysis (left) and the fraction of the overall variance attributed to the first five eigenvalues, with the first two columns, in gray, representing the first two discriminant functions (right). (PDF 1284 kb) [file 13323_2015_30_MOESM6_ESM.pdf]
