# Supplementary material for: Early modern human dispersal from Africa: genomic evidence for multiple waves of migration
Source: Investig Genet. 2015 Nov 6;6:13. doi: 10.1186/s13323-015-0030-2 (PMC4636834; doi:10.1186/s13323-015-0030-2)
Supplement: Additional file 9: — Pairwise F ST values estimated between populations. The matrix is symmetrical. (PDF 761 kb) [file 13323_2015_30_MOESM9_ESM.pdf]

FST	South_Africa	East_Africa	West_Africa	Europe	Caucasus	West_Asia	Central_Asia	North_India	South_India	East_Asia	South_Asia	Malaysia	Borneo	Sumatra	East_Indonesia	Philippine	Moluccas	Australia	New_Guinea	Fiji	Polynesia	Onge	Jehai	Mamanwa
South_Africa	0.00	0.02	0.02	0.16	0.15	0.14	0.15	0.14	0.14	0.18	0.17	0.18	0.18	0.19	0.17	0.19	0.18	0.22	0.25	0.19	0.20	0.23	0.19	0.20
East_Africa	0.02	0.00	0.01	0.13	0.12	0.12	0.13	0.12	0.12	0.16	0.15	0.15	0.16	0.16	0.15	0.16	0.16	0.19	0.22	0.16	0.18	0.20	0.17	0.17
West_Africa	0.02	0.01	0.00	0.15	0.15	0.14	0.15	0.14	0.14	0.18	0.17	0.17	0.17	0.18	0.17	0.18	0.17	0.21	0.23	0.18	0.19	0.22	0.19	0.19
Europe	0.16	0.13	0.15	0.00	0.01	0.02	0.05	0.04	0.05	0.11	0.09	0.10	0.11	0.11	0.10	0.11	0.11	0.15	0.18	0.12	0.13	0.15	0.12	0.13
Caucasus	0.15	0.12	0.15	0.01	0.00	0.01	0.05	0.03	0.04	0.10	0.08	0.09	0.10	0.10	0.09	0.10	0.10	0.15	0.18	0.11	0.12	0.15	0.11	0.12
West_Asia	0.14	0.12	0.14	0.02	0.01	0.00	0.03	0.01	0.01	0.08	0.06	0.07	0.08	0.08	0.07	0.08	0.08	0.13	0.16	0.09	0.10	0.13	0.09	0.10
Central_Asia	0.15	0.13	0.15	0.05	0.05	0.03	0.00	0.03	0.04	0.02	0.02	0.04	0.03	0.04	0.04	0.04	0.04	0.12	0.16	0.07	0.07	0.12	0.07	0.07
North_India	0.14	0.12	0.14	0.04	0.03	0.01	0.03	0.00	0.00	0.07	0.05	0.06	0.06	0.07	0.06	0.07	0.06	0.11	0.15	0.08	0.09	0.11	0.08	0.09
South_India	0.14	0.12	0.14	0.05	0.04	0.01	0.04	0.00	0.00	0.07	0.05	0.06	0.06	0.07	0.06	0.07	0.06	0.11	0.14	0.08	0.09	0.11	0.08	0.08
East_Asia	0.18	0.16	0.18	0.11	0.10	0.08	0.02	0.07	0.07	0.00	0.01	0.03	0.02	0.02	0.04	0.03	0.04	0.14	0.16	0.07	0.06	0.13	0.07	0.06
South_Asia	0.17	0.15	0.17	0.09	0.08	0.06	0.02	0.05	0.05	0.01	0.00	0.02	0.01	0.01	0.03	0.02	0.03	0.13	0.16	0.06	0.05	0.12	0.05	0.05
Malaysia	0.18	0.15	0.17	0.10	0.09	0.07	0.04	0.06	0.06	0.03	0.02	0.00	0.02	0.03	0.04	0.03	0.04	0.14	0.18	0.07	0.06	0.14	0.06	0.07
Borneo	0.18	0.16	0.17	0.11	0.10	0.08	0.03	0.06	0.06	0.02	0.01	0.02	0.00	0.01	0.03	0.02	0.03	0.13	0.16	0.07	0.05	0.13	0.06	0.06
Sumatra	0.19	0.16	0.18	0.11	0.10	0.08	0.04	0.07	0.07	0.02	0.01	0.03	0.01	0.00	0.03	0.02	0.03	0.15	0.18	0.07	0.05	0.14	0.07	0.06
East_Indonesia	0.17	0.15	0.17	0.10	0.09	0.07	0.04	0.06	0.06	0.04	0.03	0.04	0.03	0.03	0.00	0.03	0.01	0.08	0.10	0.03	0.04	0.14	0.07	0.06
Philippine	0.19	0.16	0.18	0.11	0.10	0.08	0.04	0.07	0.07	0.03	0.02	0.03	0.02	0.02	0.03	0.00	0.03	0.14	0.18	0.07	0.05	0.15	0.08	0.05
Moluccas	0.18	0.16	0.17	0.11	0.10	0.08	0.04	0.06	0.06	0.04	0.03	0.04	0.03	0.03	0.01	0.03	0.00	0.10	0.11	0.03	0.04	0.14	0.08	0.06
Australia	0.22	0.19	0.21	0.15	0.15	0.13	0.12	0.11	0.11	0.14	0.13	0.14	0.13	0.15	0.08	0.14	0.10	0.00	0.08	0.07	0.13	0.20	0.16	0.15
New_Guinea	0.25	0.22	0.23	0.18	0.18	0.16	0.16	0.15	0.14	0.16	0.16	0.18	0.16	0.18	0.10	0.18	0.11	0.08	0.00	0.07	0.15	0.23	0.20	0.18
Fiji	0.19	0.16	0.18	0.12	0.11	0.09	0.07	0.08	0.08	0.07	0.06	0.07	0.07	0.07	0.03	0.07	0.03	0.07	0.07	0.00	0.03	0.15	0.10	0.09
Polynesia	0.20	0.18	0.19	0.13	0.12	0.10	0.07	0.09	0.09	0.06	0.05	0.06	0.05	0.05	0.04	0.05	0.04	0.13	0.15	0.03	0.00	0.16	0.10	0.08
**Onge**	0.23	0.20	0.22	0.15	0.15	0.13	0.12	0.11	0.11	0.13	0.12	0.14	0.13	0.14	0.14	0.15	0.14	0.20	0.23	0.15	0.16	0.00	0.16	0.17
**Jehai**	0.19	0.17	0.19	0.12	0.11	0.09	0.07	0.08	0.08	0.07	0.05	0.06	0.06	0.07	0.07	0.08	0.08	0.16	0.20	0.10	0.10	0.16	0.00	0.10
**Mamanwa**	0.20	0.17	0.19	0.13	0.12	0.10	0.07	0.09	0.08	0.06	0.05	0.07	0.06	0.06	0.06	0.05	0.06	0.15	0.18	0.09	0.08	0.17	0.10	0.00
