# Supplementary material for: Early modern human dispersal from Africa: genomic evidence for multiple waves of migration
Source: Investig Genet. 2015 Nov 6;6:13. doi: 10.1186/s13323-015-0030-2 (PMC4636834; doi:10.1186/s13323-015-0030-2)
Supplement: Additional file 10: — Estimates of population divergence times from East, West, and South Africa. (PDF 193 kb) [file 13323_2015_30_MOESM10_ESM.pdf]

TIME	South_Africa			East_Africa			West_Africa		
	0.025	0.5	0.975	0.025	0.5	0.975	0.025	0.5	0.975
Europe	66,226	74,595	84,924	62,664	69,736	78,916	73,102	81,354	90,852
Caucasus	65,166	74,933	84,342	60,080	68,143	76,336	71,619	81,228	89,838
West_Asia	66,093	73,546	82,512	60,187	66,318	74,087	72,342	79,715	87,970
Central_Asia	70,530	76,762	87,029	65,785	71,021	80,083	77,427	83,596	93,062
North_India	72,283	77,974	88,657	65,533	70,230	79,438	78,217	83,829	93,720
South_India	64,381	69,703	78,031	59,931	64,396	71,782	70,879	76,166	83,809
East_Asia	81,840	89,638	100,743	80,536	87,432	97,802	89,783	97,477	107,621
South_Asia	76,716	85,002	95,110	73,452	80,587	89,791	84,104	92,279	101,504
Malaysia	68,379	74,544	85,294	66,345	71,622	81,344	75,427	81,433	91,098
Borneo	76,196	82,244	92,122	74,750	80,056	89,253	84,053	90,029	98,981
Sumatra	76,978	84,720	95,240	75,306	82,043	91,758	84,643	92,222	101,689
East_Indonesia	68,677	74,492	85,980	66,578	71,576	81,948	75,922	81,629	92,106
Philippine	74,563	81,547	93,021	73,171	79,248	89,786	82,217	89,053	99,441
Moluccas	67,447	73,760	83,966	66,115	71,562	80,897	74,783	80,951	90,113
**Australia**	**87,097**	**97,946**	**113,933**	**87,017**	**96,599**	**111,394**	**94,996**	**105,461**	**119,900**
**New_Guinea**	**97,168**	**106,353**	**120,861**	**98,962**	**107,204**	**121,010**	**103,534**	**112,166**	**124,690**
Fiji	71,737	78,848	87,580	71,173	77,395	85,605	79,370	86,315	94,016
Polynesia	70,269	77,397	88,039	71,230	77,531	87,551	78,186	85,110	94,553
Onge	75,400	81,853	92,406	76,885	82,572	92,571	83,702	89,904	99,017
Jehai	66,032	72,532	82,650	65,885	71,521	80,870	73,786	80,104	89,067
Mamanwa	67,458	73,580	85,147	67,671	73,012	83,689	75,252	81,200	91,540
