# Supplementary material for: Early modern human dispersal from Africa: genomic evidence for multiple waves of migration
Source: Investig Genet. 2015 Nov 6;6:13. doi: 10.1186/s13323-015-0030-2 (PMC4636834; doi:10.1186/s13323-015-0030-2)
Supplement: Additional file 11: — Population divergence time estimated on a subset of SNPs chosen to exclude the effect of an archaic introgression from Denisovan. (PDF 187 kb) [file 13323_2015_30_MOESM11_ESM.pdf]

TIME	South_Africa			East_Africa			West_Africa		
	0.025	0.5	0.975	0.025	0.5	0.975	0.025	0.5	0.975
Europe	66,439	74,835	85,197	62,892	69,990	79,204	73,357	81,638	91,169
Caucasus	65,303	75,091	84,520	60,178	68,254	76,460	71,788	81,420	90,050
West_Asia	66,348	73,829	82,830	60,456	66,614	74,419	72,613	80,013	88,299
Central_Asia	70,630	76,871	87,152	65,953	71,201	80,287	77,582	83,764	93,248
North_India	72,612	78,328	89,060	65,827	70,545	79,794	78,519	84,153	94,081
South_India	64,608	69,948	78,306	60,135	64,615	72,026	71,110	76,414	84,082
East_Asia	82,193	90,024	101,177	80,888	87,813	98,229	90,159	97,885	108,072
South_Asia	76,962	85,275	95,415	73,752	80,916	90,158	84,389	92,591	101,848
Malaysia	68,561	74,742	85,521	66,551	71,844	81,597	75,623	81,646	91,336
Borneo	76,419	82,485	92,392	75,000	80,324	89,551	84,283	90,274	99,251
Sumatra	77,292	85,065	95,628	75,650	82,418	92,177	84,990	92,600	102,106
East_Indonesia	68,821	74,649	86,161	66,771	71,783	82,186	76,159	81,884	92,394
Philippine	74,840	81,850	93,367	73,550	79,658	90,251	82,590	89,457	99,892
Moluccas	67,705	74,043	84,288	66,378	71,847	81,219	75,142	81,340	90,546
**Australia**	**87,110**	**97,961**	**113,951**	**87,057**	**96,644**	**111,446**	**95,130**	**105,609**	**120,069**
**New_Guinea**	**97,273**	**106,468**	**120,992**	**99,053**	**107,302**	**121,121**	**103,811**	**112,466**	**125,023**
Fiji	71,879	79,003	87,753	71,344	77,580	85,810	79,569	86,531	94,252
Polynesia	70,479	77,628	88,303	71,447	77,768	87,819	78,417	85,362	94,833
Onge	75,670	82,147	92,738	77,173	82,881	92,918	83,983	90,206	99,349
Jehai	66,223	72,742	82,889	66,108	71,763	81,143	73,966	80,300	89,284
Mamanwa	67,531	73,660	85,240	67,779	73,129	83,822	75,354	81,310	91,664
